# Soluble CD14 promotes Th17 expansion and differentiation through gamma-aminobutyric acid and expands non-canonical innate lymphoid cells

**DOI:** 10.1093/pnasnexus/pgaf406

**Published:** 2025-12-29

**Authors:** Shima Shahbaz, Amirhossein Rahmati, Hussain Syed, Shokrollah Elahi

**Affiliations:** Division of Foundational Sciences, Mike Petryk School of Dentistry, Faculty of Medicine and Dentistry, University of Alberta, Edmonton, AB, Canada T6G 2E1; Division of Foundational Sciences, Mike Petryk School of Dentistry, Faculty of Medicine and Dentistry, University of Alberta, Edmonton, AB, Canada T6G 2E1; Division of Gastroenterology, Department of Medicine, Faculty of Medicine and Dentistry, University of Alberta, Edmonton, AB, Canada T6G 2E1; Division of Foundational Sciences, Mike Petryk School of Dentistry, Faculty of Medicine and Dentistry, University of Alberta, Edmonton, AB, Canada T6G 2E1; Li Ka Shing Institute of Virology, Faculty of Medicine and Dentistry, University of Alberta, Edmonton, AB, Canada T6G 2R3; Women and Children Health Research Institute, Faculty of Medicine and Dentistry, University of Alberta, Edmonton, AB, Canada T6G 1C9; Cancer Research Institute of Northern Alberta, Faculty of Medicine and Dentistry, University of Alberta, Edmonton, AB, Canada T6G 2E1; Glycomics Institute of Alberta, University of Alberta, Edmonton, AB, Canada T6G 2G2; Alberta Transplant Institute, Faculty of Medicine and Dentistry, University of Alberta, Edmonton, AB, Canada T6G 2E1

**Keywords:** innate lymphoid cells, gamma-aminobutryic acid (GABA), 4-aminobutyrate, SARS-CoV-2, long COVID

## Abstract

Interleukin-17 (IL-17) plays a central role in the pathogenesis of various autoimmune diseases. Soluble CD14 (sCD14), a marker of innate immune activation, is elevated in several inflammatory conditions. However, its influence on IL-17 production and the differentiation of Th17 cells remains poorly understood. We found that sCD14 enhances Th17-associated cytokine production and up-regulates critical transcription factors such as STAT3 and RORC. Notably, sCD14's effect on Th17 polarization was mediated indirectly through autologous sCD14-treated peripheral blood mononuclear cell (PBMC) supernatant (sCD14-PBMC-Sup). Additionally, we identified a distinct cytokine profile enriched for pro-inflammatory cytokines and chemokines in sCD14-treated T cells, further reinforcing the Th17-promoting role of sCD14. Interestingly, gamma-aminobutyric acid (GABA), a metabolite elevated in sCD14-treated monocytes, was identified as a potential contributor to Th17 polarization. GABA supplementation in T-cell cultures enhanced IL-17A secretion, indicating its role as a signaling molecule in T-cell differentiation. Our findings also revealed the expansion of innate lymphoid cell (ILC)2/3-like cells in T-cell cultures exposed to sCD14-PBMC-Sup and GABA, highlighting the potential role of monocytes in Th17-mediated immunity. Furthermore, while sCD14 promoted Th17 polarization, it simultaneously impaired T-cell activation and proliferation, suggesting an immunosuppressive effect mediated by soluble factors released from monocytes. These results underscore the dual role of sCD14 in modulating T-cell responses, promoting Th17 differentiation while suppressing T-cell effector functions. This study identifies a previously unrecognized role for sCD14 in promoting Th17 induction, highlighting its contribution to immune regulation and its potential as a therapeutic target in Th17-driven autoimmune conditions.

Classification: Immunology

Significance Statement Soluble CD14 (sCD14) is well known for its role in innate immunity; however, only a single study has demonstrated its inhibitory effects on T cells. Our study reveals a dual role for sCD14 in human immunity: while it suppresses T-cell activation and proliferation, it paradoxically promotes Th17 differentiation and innate lymphoid cell expansion through monocyte-derived soluble factors and the metabolite gamma-aminobutyric acid. These findings uncover a previously unrecognized immunomodulatory function of sCD14 in shaping adaptive immune responses, particularly Th17 polarization, and suggest a novel link between metabolic signaling and T-cell fate. Understanding this pathway opens new therapeutic opportunities for modulating Th17-driven inflammation in autoimmune and chronic inflammatory diseases.

## Introduction

Th17 cells, as the major source of interleukin-17 (IL-17)-producing T cells, play essential roles against fungal and bacterial infections and promote mucosal immunity (1). IL-17 family consists of six related cytokines, IL-17A through IL-17F, each with context-dependent biological roles (2). Despite their protective role, Th17 cells are associated with the pathogenesis of autoimmune diseases, cancer, and various inflammatory conditions (2–4). Notably, despite extensive investigations, the role of differential cues in promoting Th17 cells in pathological conditions remains an area of intense interest.

The differentiation of Th17-effector cells can be achieved in vitro by T cell receptor (TCR) stimulation of naive T cells in the presence of cytokines, such as IL-6 or IL-21, along with transforming growth factor-beta (TGF-β), which effectively induce the lineage-specific transcription factor (TF), retinoic acid receptor–related orphan receptor (RORγt) (5). IL-6 is recognized as a key inflammatory cytokine that, in combination with TGF-β, drives Th17-cell differentiation (6, 7). When IL-6 binds to its receptor, it activates the STAT3, which subsequently enhances the expression of RORγt (7). IL-21 can also activate the STAT3 pathway as an alternative cytokine. In combination with TGF-β, IL-21 induces the expression of RORγt and drives Th17-cell differentiation, functioning independently of IL-6 (8). Other cytokine, such as IL-1, signaling has been reported to be crucial for early Th17-mediated inflammation in experimental autoimmune encephalomyelitis (EAE) (9). Additionally, IL-23 activates the STAT3 pathway to maintain the Th17 lineage, though it does not drive the initial commitment of naive T cells to the Th17 fate (10).

Recent evidence has revealed that serum amyloid A proteins can also induce Th17 differentiation independently of TGF-β (11). Similarly, prostaglandin E2 with IL-1β and IL-23 in a synergistic manner drives RORγt expression (12). Additionally, hypoxia-inducible factor 1-alpha (HIF-1α)-dependent glycolysis pathway plays a crucial role in Th17 differentiation, suggesting a selective reliance on glucose and amino acid metabolism (13). Similarly, Th17 cells depend on acetyl-CoA carboxylase 1–mediated fatty acid synthesis for their induction (14). Moreover, amino acid metabolism—particularly glutaminolysis, the conversion of glutamine to glutamate—is crucial for Th17-cell development (15).

Four-aminobutanoate (gamma-aminobutyric acid, GABA) and its receptors (GABA-Rs) are well-known for their roles in neurotransmission and neurodevelopment within the central nervous system (16). GABA is synthesized within cells via the decarboxylation of glutamate by the enzymes glutamic acid decarboxylase 65 (GAD65) and glutamic acid decarboxylase 67 (GAD67). In recent years, studies have shown that GABA is produced and secreted by B cells and natural killer (NK) cells (17, 18). Yet, its effects on T cells remain somewhat controversial. While some studies indicate that GABA inhibits T-cell effector functions (19), others suggest that GABA enhances IL-17 expression in the intestinal tissues in an animal infection model (20).

Subsequent studies have revealed that a variety of immune cell types, including type 3 innate lymphoid cells (ILC3s), γδ T cells, and NKT cells, also produce IL-17 (2). However, production of IL-17 by myeloid cells such as neutrophils and microglia remains controversial (21). ILCs are classified into five subsets based on their transcriptional factors and cytokine profiles (22); however, we discuss three major subsets. Group 1 ILCs (ILC1s) typically rely on the TF T-bet for their development and produce interferon-gamma (IFN-γ) as their primary effector cytokine (23). The helper ILCs include group 2 ILCs (ILC2s), which depend on GATA-3, and group 3 ILCs (ILC3s), which require RORγt for their development (24, 25). However, ILCs exhibit significant plasticity, allowing them to alter their phenotype and function according to the microenvironmental milieu. For example, when exposed to IL-12, ILC2s, and ILC3s lose the expression of GATA-3 and RORγt, respectively, while acquiring characteristics of typical ILC1s (26–28). Therefore, identifying surrogate markers that can influence the plasticity of ILC subsets remains a significant challenge in the field.

Membrane-bound CD14 (mCD14) is a glycoprotein that is widely expressed in both immune and nonimmune cells. In immune cells, it is mainly expressed by myeloid lineage cells, including granulocytes, monocytes, macrophages, and dendritic cells (DCs) (29, 30). CD14 is located in lipid rafts and anchored to the outer layer of the plasma membrane by a glycosylphosphatidylinositol (GPI) moiety (29). It primarily acts as a co-receptor for Toll-like receptors (TLRs), particularly TLR4, which triggers the activation of MyD88- and TIR-domain-containing adapter-inducing interferon-β (TRIF)-dependent pathways (31). These pathways mediate the secretion of pro-inflammatory cytokines and type I IFNs, respectively (32). Additionally, CD14 activates TLR4-independent signaling pathways, such as nuclear factor of activated T cells (NFAT) signaling in DCs and noncanonical inflammasome pathways, which lead to the activation of pro-apoptotic genes or the secretion of IL-1β and IL-18, respectively (33). Beyond its immune-related properties, soluble CD14 (sCD14) plays important roles in nonimmune processes, including the regulating of insulin resistance in obesity (34).

sCD14 can be released from the cell surface through several mechanisms, including the removal of its C-terminal residue, which is critical for its attachment to the GPI anchor, bypassing posttranslational modifications, and phospholipase D-mediated cleavage of the GPI anchor (35). While other cells, such as hepatocytes and gastrointestinal epithelial cells, can secrete sCD14 (36, 37), human monocytes are its primary source, particularly upon activation (38). Elevated plasma levels of sCD14 have been observed in various inflammatory conditions and viral infections (e.g. HIV and SARS-CoV-2) (39–41). Therefore, circulating sCD14 is mainly associated with monocyte activation and regarded as damage-associated molecular pattern or acute-phase protein (42, 43). However, there are limited studies on its role on T cells. A quarter century ago, the inhibitory effects of sCD14 on T-cell proliferation and effector functions, such as IL-2, IL-4, and IFN-γ production, were reported (44). Another study has reported that IL-1β-primed DCs via the expression of CD14 promote IL-17 production in memory CD4 T cells (45). As such, further studies are urgently needed to determine whether sCD14 inhibits T-cell effector functions or promotes their differentiation.

In this study, we demonstrate that sCD14 promotes the expansion of IL-17-secreting T cells and induces the polarization of both naive CD4 and CD8 T cells into IL-17-producing cells. Intriguingly, we found that sCD14 does not directly promote T-cell differentiation but instead, through interaction with monocytes enhances GABA production, which subsequently influences T-cell differentiation. Additionally, we show that sCD14 facilitates the expansion of IL-17F-producing ILC2 subset. Our findings reveal a novel mechanism by which sCD14 contributes to the differentiation of naive T cells into IL-17-secreting subsets.

## Materials and methods

### Ethics statement

This study was approved by the Human Research Ethics Board at the University of Alberta (Pro00099502 and Pro00063463) with informed written consent being obtained from all study participants. For our studies, we collected blood samples from 35 healthy individuals (HCs) with a median age 49.5 ± 12.1, 18 females and 17 males who were seronegative for HIV, hepatitis C virus, and hepatitis B virus, and had no other co-morbidities. Plasma samples from a cohort of 34 long COVID (LC) patients with myalgic encephalomyelitis or chronic fatigue syndrome (ME/CFS) (46, 47) (median age 48 ± 9.8, 25 females and nine males) and a cohort of 50 acute COVID-19 patients (48) (median age 62 ± 11, 30 females and 20 males) were used for sCD14 and IL-17A enzyme-linked immunosorbent assay (ELISA) assays.

### Cell isolation and culture

Peripheral blood mononuclear cells (PBMCs) were isolated by gradient separation using Ficoll-Paque PREMIUM (GEU Healthcare, Chicago, IL, United States) and cultured in RPMI 1640 (Sigma-Aldrich) supplemented with 10% fetal bovine serum (FBS) (Sigma-Aldrich) and 1% penicillin/streptomycin (Sigma-Aldrich). T cells were isolated using a human T-cell isolation kit (Stem Cell Technology, Cat# 17951) with a purity of >95%, as we previously reported (49) (Fig. S1A). Naive CD4 and CD8 T cells were isolated using human naive CD4 and CD8 isolated kits, respectively (Stem Cell Technology, Cat# 19555 and Cat# 19258), with >95% purity for both isolated naive CD4 and CD8 T cells (Fig. S1B and C). Total PBMCs or isolated T cells were cultured with recombinant human CD14 protein (R&D, Cat# 383-CD-050) overnight and then stimulated the following day with anti-human CD3 (UCHT1, 3 μg/mL)/CD28 (CD28.2, 1 μg/mL). In some experiments, supernatants from PBMCs stimulated with sCD14 for 16–18 h were collected and added to freshly isolated autologous T cells. In parallel, supernatants from PBMCs cultured without sCD14 were used as controls and added to autologous T cells. While the culture supernatants were collected for ELISA assays, RNA from the cell pellets was isolated for qPCR and RNA sequencing. For Th17 conditioning, isolated T cells were stimulated with anti-CD3/CD28 antibodies in the presence of human IL-6 (30 ng/mL; Stem Cell Technology, Cat# 78050.1), IL-23 (30 ng/mL; Stem Cell Technology, Cat# 78050.1), IL-1β (20 ng/mL; Stem Cell Technology, Cat# 78143), TGF-β (2.5 ng/mL; BioLegend, Cat# 580704), anti-human IL-4 (2.5 μg/mL; Miltenyi Biotec, Cat# 130-095-753), and anti-human IFN-γ (1 μg/mL; Miltenyi Biotec, Cat# 130-095-743). For proliferation assay, PBMCs were labeled with CellTrace CFSE and stimulated with anti-CD3 (2 μg/mL) for 96 h in the presence or absence of sCD14.

To evaluate the effects of sCD14 on the proliferation of the Th17 subset, total PBMCs were stimulated with anti-CD3 (2 μg/mL) for 96 h. Six hours before the end of the assay, cells were restimulated with phorbol myristate acetate (PMA)/inomycin (10 ng/mL) in the presence of a Golgi blocker. Because of its higher yield, IL-17F was used as the Th17 marker among proliferating cells. Monocytes were isolated using EasySep Human Monocyte Enrichment Kit (Cat# 19059) with ∼95% purity (Fig. S1D) and cultured with sCD14 overnight. The supernatant from cultured monocytes was also collected for multiplex ELISA and/or metabolomic analyses.

### Flow cytometry analysis

The fluorochrome-conjugated antibodies were purchased mainly from BD Biosciences, Thermo Fisher Scientific, and BioLegend. The following human antibodies were used for the study: anti-CD3 (SK7), anti-CD4 (RPA-T4), anti-CD8 (RPA-T8), anti-TNF-α (MAB11), anti-IFN-γ (45.B3), anti-IL-2 (MQ1-17H12), anti-CD45RO (UCHL1), anti-CD45RA (HL100), anti-CD25 (M-A251), anti-CD69 (FN50), anti-HLA-DR (LN3), anti-IL-17F (SHLR17), anti-CD11b (ICRF44), anti-CD33 (P67.6), anti-CD117 (YB5.B8), anti-CD294 (BM16), anti-CD7 (4H9), anti-CD19 (HIB19), anti-CD45 (HI30), anti-CCR7 (2-L1-A), and anti-CD127 (HIL-7R-M21).

Apoptotic assay was performed using the PE Annexin V Apoptosis Detection Kit I (BD Biosciences) and cell viability was assessed using LIVE/DEAD Kit (Life Technologies) (50). Surface staining and intracytoplasmic cytokine staining were performed according to our previous protocols (51, 52). The stained cells were fixed in paraformaldehyde (4%), and the data were acquired on an LSR Fortessa-SORP flow cytometer (BD Biosciences) and analyzed with FlowJo software (version 10).

### Library construction and sequencing

We used Direct-zol RNA MicroPrep kit (Zymo Research) for the extraction of total RNA. Libraries were made (100 ng RNA) using the TruSeq RNA Library Prep Kit v2 (Illumina, San Diego, CA, United States). Briefly, oligo dTs conjugated to paramagnetic beads were used to pull down polyadenylated mRNAs and nonpolyadenylated transcripts were removed using sequential EtOH washes. First- and second-strand cDNAs made from chemically fragmented recovered mRNAs were blunted and A-tailed by T-A ligation. Finally, 12 cycles of PCR were used to incorporate Illumina adapters containing multiplexing barcodes, with sequencing performed on a HiSeq 2500 instrument (53). Library sequencing was performed on the NovoSeq X platform (Illumina) by Novogene (Sacramento, CA, United States).

### Bioinformatic analyses

We used Kallisto with 100 permutations and bias correction for fragment alignment to the human cDNA database (GRCh38). Tximport (R version 4.4.0) was used to generate count and transcripts per million (TPM) matrices for the genes with differentially expressed genes (DEGs; corrected *P*-value (*P*_adj_) < 0.05 and −1 < log_2_-fold change (FC) > + 1) defined using the DESeq2 R package (R version 4.4.0) as we have previously reported (53). Heat map, volcano, and box plots were generated using R scripts. Transcript pathway analysis was performed using decoupleR package.

### Single-cell RNA sequencing

T cells from each condition were processed for single-cell RNA sequencing (scRNA-seq) using the 10× Genomics Chromium Controller (PN-1000121) using the 10× Genomics Chromium Next GEM Single Cell 3′ GEM, Library and Gel Bead Kit v3.1 (PN-1000121), following the manufacturer's instructions and sequenced on the Illumina NovaSeq × Plus 25B PE150 (Novogene). Droplet-based sequencing data from 10× Genomics were aligned and quantified against the GRCh38 human reference genome using Cell Ranger v8.0. The resulting filtered count matrix generated by Cell Ranger was subsequently used for downstream analysis.

### Quality control and clustering

All the quality control was performed using Seurat 5.1.0 in the R statistical environment (v4.4.0) (54). We created the Seurat objects to include only genes expressing in a minimum of three cells and cells that expressed a minimum of 200 genes using the CreateSeuratObject function. The subset function was used further to remove cells with >4,000 gene counts (dead cells and doublets or multiplets) and cells with high percentages (>5%) of mitochondrial genes. Datasets were merged with the merge function and normalized with the SCTransform function according to the binomial regression model (55).

Dimensionality reduction was performed successively using RunPCA, FindNeighbours, and FindClusters functions while retaining 40 PCs, as determined by a PCA elbow plot. The final resolution of 0.5 was selected based on the clustering tree generated from the Clustree package (56). The differentially expressed genes (DEGs) in each cluster were calculated in Seurat using the FindAllMarkers function (min. pct = 0.25, log-FC threshold = 0.25, *P* < 0.05), and the average gene expression values were plotted in Seurat using the DoHeatmap function. Density plots and dot plots were generated using the plot density function of the Nebulosa package (v1.15.0) and DotPlot function, respectively (57).

### Gene expression analysis

The RNA was isolated from approximately 2 × 10^6^ T cells using the Direct-zol RNA MicroPrep kit (Zymo Research) and cDNA (100 ng) for mRNA expression was synthesized using the QuantiTect Rev. Transcription Kit (Qiagen), as described previously (52). Expression of IL17A mRNA was analyzed using QuantiTect SYBR Green Primer Assay (Qiagen) and CFX96 Touch Real-Time PCR Detection System (Bio-Rad). Beta-2-microglobulin was used as a reference gene for gene expression, and the data were analyzed using the 2^−ddCT^ method, as we reported elsewhere (58).

### In vitro cytokine and chemokine measurements

The concentration of cytokines/chemokines in culture supernatants was measured by Mesoscale Discovery (MSD) kits, with the data acquired on a V-plex Sector Imager 2400 plate reader and analyzed using the MSD workbench 3.0 software (MSD, Rockville, MD, United States), as reported elsewhere (41, 59). The concentrations of IL-17A and sCD14 were measured by DuoSet ELISA kit (R&D systems, Cat# DY317 and Cat# DY383-05, respectively) following the manufacturer's protocols. IL-17 level was also measured by performing ELISpot assay (Mabtech, Cat# 3523-2A). Isolated T cells were cultured at the concentration 1 × 10^5^ cells per well in the presence or absence of supernatant from autologous PBMCs stimulated with sCD14 and ImmunoSpot (CTL ImmunoSpot Analyzer from Cellular Technology Ltd) was employed to measure IL-17A/F-secreting cells according to our methods (60, 61).

### Metabolomic profiling

Samples were randomized by measuring the total metabolite concentration in each sample using the proprietary metabolome quantification kit (Nova Medical Testing Inc., Cat# NMT-6001-KT) to adjust the samples to the same concentration of 1.2 mM. The metabolomic analysis was performed according to the Chemical Isotope Labeling LC-MS protocol (62). Postacquisition normalization of the data was performed by Ratio of Total Useful Signal, which is the ratio of the sum of all useful ^12^C-peaks over the sum of all useful ^13^C-peaks (40).

### Statistical analysis

Statistical analysis was performed using GraphPad Prism 10 (GraphPad Software, Inc.). Mann–Whitney *U* test was used to compare unpaired, non-normally distributed data sets. Data are presented as median with interquartile range. For comparisons involving more than two groups, one-way ANOVA followed by Tukey's post hoc test was used. Paired comparisons were analyzed using the Wilcoxon matched-pairs signed-rank test.

## Results

### sCD14 suppresses T-cell effector functions

**Fig. 1.**
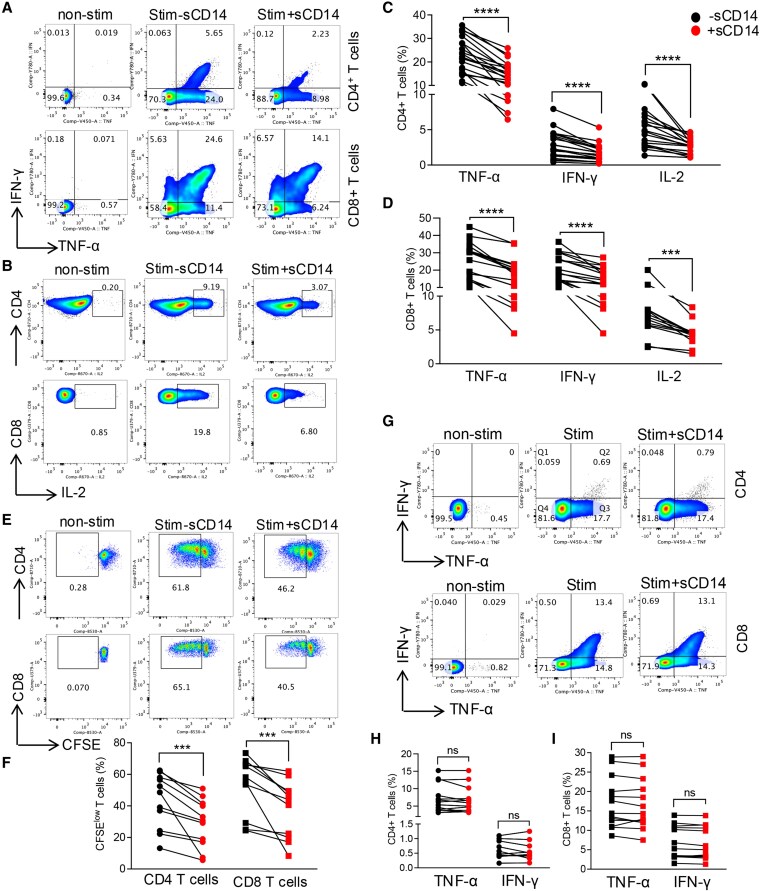
sCD14 impairs cytokine production and T-cell proliferation within PBMCs. A) Representative flow cytometry plots, and B) cumulative data of TNF-α, IFN-γ, and IL-2 expression in C) CD4 and D) CD8 T cells after treatment of PBMCs with anti-CD3/CD28 in the presence and absence of sCD14 (1 μg/mL). E) Representative flow cytometry plots, and F) cumulative data of CD4 and CD8 T-cell proliferation in the presence and absence sCD14. G) Representative plots, and cumulative data of IFN-γ and TNF-α expression in H) CD4, and I) CD8 T cells. *P* values were calculated using Wilcoxon matched-pairs two-tailed t test; ***<0.001, ****<0.0001. Each dot represents sample from a study subject.

Considering the elevation of sCD14 in various pathological conditions (39–41), we decided to determine its effects on T-cell effector functions. Briefly, PBMCs were stimulated with anti-CD3/CD28 in the absence or presence of a physiological concentration of sCD14 (1 μg/mL) overnight (40). We found that sCD14 significantly inhibited TNF-α, IFN-γ, and IL-2 production by both CD4 and CD8 T cells (Figs. 1A–D and S1E). Similarly, we observed that sCD14 suppressed T-cell proliferation (Fig. 1E and F). Additionally, sCD14 significantly reduced the expression of activation markers CD25 and CD69 in both T-cell subsets (Fig. S2A–F), without altering their survival rate (Fig. S2G and H).

As an alternative pathway, we hypothesized that sCD14 may mediate its suppressive effects through the conversion of monocytes into myeloid-derived suppressor cells (MDSCs). However, we observed that sCD14-activated monocytes (e.g. increased HLA-DR expression) and reduced the subset of monocytic-MDSCs (Fig. S2I).

### sCD14 does not exhibit direct T-cell suppression

We found that sCD14 did not exhibit any immunosuppressive effects on cytokine production by isolated T cells (Fig. 1G–I). These findings suggest that sCD14 mediates its effects indirectly, possibly through interaction with other components of PBMCs.

To explore this further, we collected the culture supernatant from PBMCs treated overnight with sCD14 (sCD14-PBMC-Sup) and assessed its effects on the functionality of isolated autologous T cells. Interestingly, we observed that the sCD14-PBMC-Sup, but not sCD14, significantly impaired T-cell effector function (e.g. TNF-α production) in both CD4 and CD8 T cells (Fig. S3A–C). Notably, the sCD14-PBMC-Sup produced the same effects as sCD14 on PBMCs (Figs. S3A and 3D and E). These findings indicate that sCD14 interacts and/or influences other components of PBMCs, leading to T-cell suppression.

### sCD14 polarizes T cells toward a Th17 phenotype

**Fig. 2.**
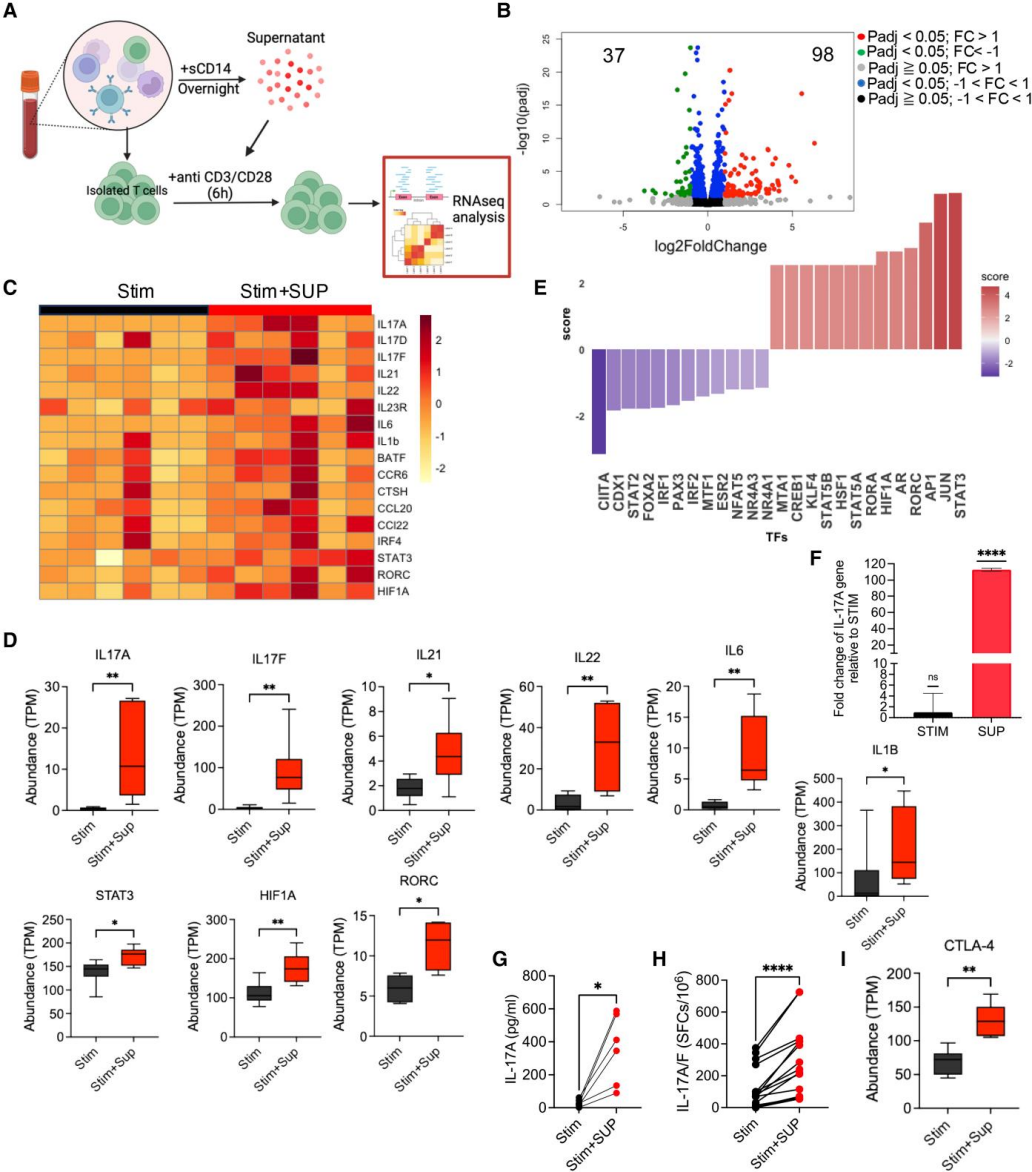
The supernatant of sCD14-treated PBMCs promotes Th17 phenotype. A) The schematic plot of the experimental design. B) Volcano plot of differentially expressed genes in isolated T cells stimulated with anti-CD3/CD28, with and without supernatant from sCD14-treated PBMCs. C) Heat map comparing differentially expressed genes associated with Th17 cells in isolated T cells stimulated with anti-CD3/CD28 plus supernatant from unstimulated autologous PBMCs, and treated with the supernatant from sCD14-treated PBMCs. D) Abundance (TPM) of various genes associated with Th17 cells in isolated T cells, with or without treatment with the supernatant from sCD14-treated PBMCs. E) Bar plot showing the up- and down-regulated transcriptional factors in isolated T cells after treatment with the supernatant from sCD14-treated PBMCs. F) Fold change in IL-17A gene expression in isolated T cells after treatment with the supernatant from sCD14-treated PBMCs. G) IL-17A levels in culture supernatants of isolated T cells treated with the supernatant from sCD14-treated PBMCs, as quantified by ELISA. H) Isolated T cells (1 × 10^5^) were stimulated in the presence of the supernatant from sCD14-treated PBMCs, and IL-17A-secreting cells were visualized and enumerated by ELISpot. I) Box plot showing the expression of CTLA-4 gene in T cells stimulated with anti-CD3/CD28, in the presence and absence of the supernatant from sCD14-treated PBMCs. *P* values were calculated using two-tailed Mann–Whitney *U* test (D, F, I) and Wilcoxon matched-pairs signed-rank tests (G, H); *P < 0.05, **<0.01, ****<0.0001. Each dot represents sample from a study subject.

We collected sCD14-PBMC-Sup following overnight culture and incubated with autologous T cells, which were activated with anti-CD3/CD28 for 6 h. This was followed by RNA isolation and bulk RNA-seq of PBMCs from six study subjects per condition (Fig. 2A). RNA-seq analyses revealed the up-regulation of 98 genes and down-regulation of 37 genes in T cells treated with sCD14-PBMC-Sup compared to the control groups that were treated with autologous culture supernatant without sCD14 (Fig. 2B).

More in-depth analysis revealed a significant up-regulation of genes involved in the differentiation and function of Th17 cells, including IL17A, IL17F, IL21, IL22, IL6, IL1B, STAT3, HIF1A, and RORC (Fig. 2C and D). However, despite this elevation, the difference did not reach a significant level for IL17D, IL23R, BATF, CCR6, CCL20, and CCL22 (Fig. S3F). TF analysis using decoupleR also confirmed the up-regulation of TFs, such as STAT3, RORA, RORC, and HIF1A, which are known to be associated with Th17 cell induction (Figs. 2E and S4A–C). However, we did not observe significant changes in the expression of genes associated with Th1, Th2, or Treg subsets (Fig. S4D–F). Additionally, we confirmed that sCD14-PBMC-Sup up-regulates IL-17A mRNA in autologous T cells using qPCR (Fig. 2F). To further validate these findings, we measured IL-17A secretion from sCD14-PBMC-Sup-treated T cells using both ELISA and ELISpot assays (Fig. 2G and H). However, we did not observe a significant reduction in TNF-α and IFN-γ genes in T cells treated with the sCD14-PBMC-Sup for 6 h (Fig. S4G and H). Notably, we found that CTLA-4 was the only significantly up-regulated co-inhibitory receptor gene when T cells were treated with sCD14-PBMC-Sup (Fig. 2I), which was confirmed by qPCR (Fig. S5A). Overall, our results suggest that sCD14-PBMC-Sup either differentiates naive T cells into Th17 cells or expands Th17 cells in our cell culture system.

### sCD14 expands Th17 cells and polarizes both naive CD4 and CD8 T cells toward a Th17 phenotype

**Fig. 3.**
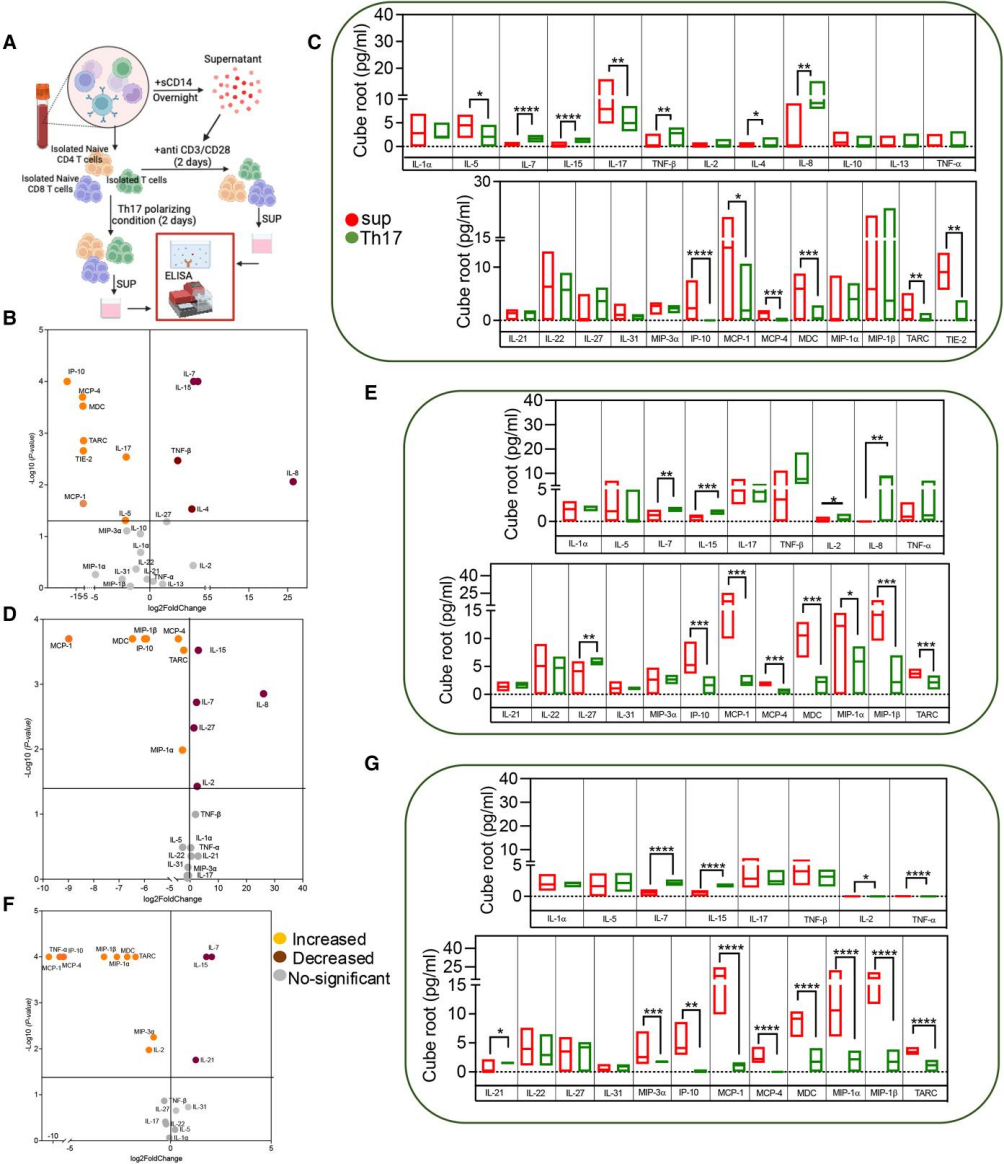
Supernatant of sCD14-treated PBMCs results in both the expansion of Th17 and polarization of naive CD4 and CD8 T cells toward a Th17 phenotype. A) Schematic flow of the experimental design. B) Volcano plot and C) bar plots illustrating the magnitude and significance of the differences in cytokine/chemokine concentrations in the supernatant of total T cells stimulated with anti-CD3/CD28 in the presence of the supernatant from sCD14-treated PBMCs or Th17-polarizing condition. D) Volcano plot and E) bar plots illustrating the magnitude and significance of the differences in cytokine/chemokine concentrations in the supernatant of naive CD4 T cells stimulated with anti-CD3/CD28 in the presence of the supernatant from sCD14-treated PBMCs or Th17-polarizing condition. F) Volcano plot and G) bar plots illustrating the magnitude and significance of differences in cytokine/chemokine concentrations in the supernatant of naive CD8 T cells stimulated with anti-CD3/CD28 in the presence of the supernatant from sCD14-treated PBMCs or Th17-polarizing conditions. *P < 0.05, **<0.01, ***<0.001, ****<0.0001.

We first demonstrated that Th17-polarizing condition induces a robust Th17 phenotype by measuring Th17-related cytokines after culturing total T cells for 48 h (Fig. S5B). Next, we isolated T cells from HCs (*n* = 8) and cultured them for 48 h with autologous sCD14-PBMC-Sup or under Th17-polarizing conditions (Fig. 3A). Additionally, to distinguish whether sCD14 promotes the expansion of existing Th17 cells in total T cells or polarizes naive T cells toward a Th17 phenotype, we isolated autologous naive CD4 and CD8 T cells and stimulated them for 48 h in the presence of sCD14-PBMC-Sup or Th17-polarizing conditions (Figs. 3A and S1B and C). Culture supernatants from different conditions were subjected to multiplex ELISA (Fig. 3A). First, we measured cytokine levels in sCD14-PBMC-Sup from each individual sample to assess the cytokine milieu of PBMCs exposed to sCD14 overnight compared with supernatants from cultures without sCD14. Among the quantified cytokines and chemokines, we observed significantly elevated levels of IL-1α, TNF-α, Tie-2, MCP-1, MIP-1β, and IL-8 (Fig. S5D). Cytokines and chemokines that were undetectable were excluded from the analysis. Notably, only sCD14-PBMC-Sup but not those from PBMCs cultured without sCD14 promoted Th17-related cytokines when total autologous T cells were treated for 48 h (Fig. S6A).

We found that total T cells treated with sCD14-PBMC-Sup, compared to T cells treated with Th17-polarizing conditions, produced significantly higher levels of IL-5, IL-17A, IP-10, MCP-1, MCP-4, MDC, TARC, and TIE-2 (Fig. 3B and C). In contrast, we detected significantly lower levels of IL-7, IL-15, TNF-β, IL-4, and IL-8 (Fig. 3B and C). Intriguingly, we observed that both naive CD4 and CD8 T cells secreted comparable levels of IL-17A in the presence of sCD14-PBMC-Sup and IL-17 conditioning media (Fig. 3D–G). This observation suggests that the sCD14-PBMC-Sup not only expands Th17 cells but also polarizes both naive T cells toward a Th17 phenotype.

When we compared the cytokine profile of naive CD4 T cells treated with sCD14-PBMC-Sup with that of naive CD4 T cells treated with the Th17-polarizing condition, we found significantly higher levels of MCP-4, MDC, TARC, MIP-1α, MIP-1β, IP-10, and MCP-1, very similar to what was observed in sCD14-PBMC-Sup-treated total T cells (Fig. 3D and E). In contrast, naive CD4 T cells treated with Th17-polarizing conditions secreted higher levels of IL-7, IL-15, IL-27, IL-8, and IL-2, which was somewhat similar to cytokines secreted by total T cells treated under Th17-polarizing conditions (Fig. 3D and E).

Additionally, a comparison of naive CD8 T cells treated with sCD14-PBMC-Sup with those treated with Th17-polarizing condition revealed significantly higher levels of MCP-4, MDC, TARC, MCP-1, TNF-α, MIP-1α, MIP-1β, MIP-3α, IP-10, and IL-2 (Fig. 3F and G). In contrast, naive CD8 T cells treated with Th17-polarizing conditions secreted significantly higher levels of IL-7, IL-15, and IL-21 (Fig. 3F and G). Moreover, we found that sCD14 promoted the proliferation of both IL-17F^+^CD4^+^ and CD8^+^ T cells after 96 h culture of PBMCs (Fig. S6B).

sCD14-PBMC-Sup not only expands Th17 cells but also induces the generation of Th17 cells from both naive CD4 and CD8 T cells. Additionally, we consistently observed higher secretion of MCP-1, MCP-4, IP-10, TARC, and MDC, but lower levels of IL-7 and IL-15 in total T cells and naive T cells treated with sCD14-PBMC-Sup compared to those treated under Th17-polarizing condition.

### sCD14 induces GABA secretion by monocytes

**Fig. 4.**
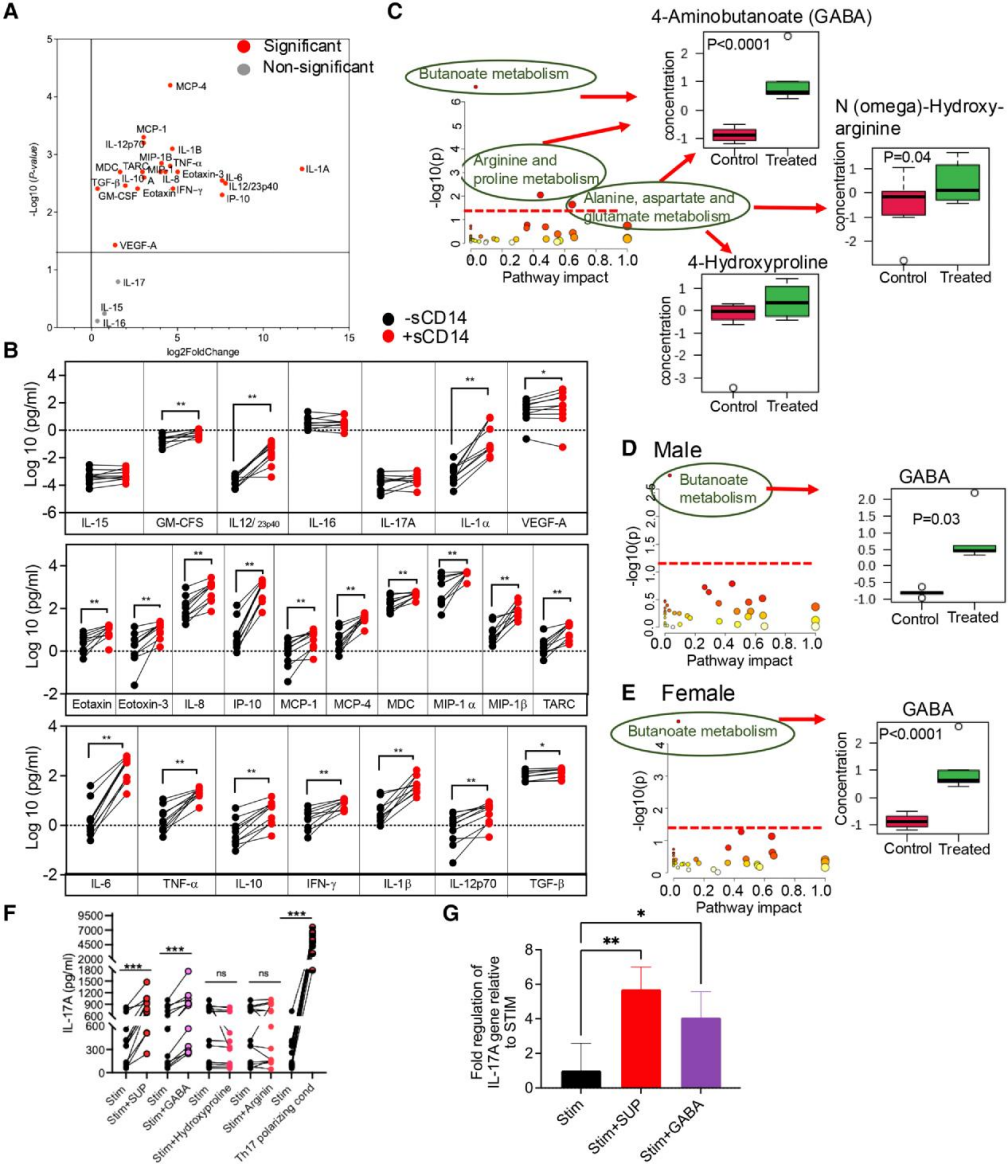
Increased GABA in the supernatant of sCD14-treated monocytes promotes IL-17A secretion. A) Volcano plot illustrating the magnitude and significance of differences in cytokine/chemokine concentrations in the supernatant of isolated monocytes from HCs (*n* = 10) with and without overnight treatment with sCD14. B) Normalized and calculated concentrations of shown cytokines in the supernatant of isolated monocytes from HCs with and without overnight treatment with sCD14. C) Metabolic pathway enrichment analysis plot showing significantly altered pathways in the supernatant of monocytes isolated from 12 HCs (six males, six females) after treatment with sCD14, with box plots showing the metabolites altered in each pathway. D) Metabolic pathway enrichment analysis plot showing significantly altered pathways in the supernatant of monocytes isolated from male HCs (*n* = 6) and E) female HCs (*n* = 6) after treatment with sCD14, with box plots showing the metabolites altered in each pathway. F) Culture supernatant concentrations of IL-17A in isolated T cells treated with the supernatant from sCD14-treated PBMCs, GABA, hydroxyproline, arginine, and Th17-polarizing conditions, as quantified by ELISA. G) Fold change in IL17A gene expression in isolated T cells after treatment with the supernatant from sCD14-treated PBMCs and GABA. *P* values were calculated using two-tailed Mann–Whitney *U* test (C, D, E), Kruskal–Wallis analysis with Dunn's multiple comparisons test (G); * *P* < 0.05, **<0.01, ***<0.001. Each dot represents sample from a human study subject.

Given that sCD14 interacts with monocytes (63, 64), we investigated whether sCD14 induces cytokines associated with Th17 differentiation. To explore this, we isolated monocytes from HCs (*n* = 10) and treated them overnight for 48 h with sCD14 (Fig. S1D). We found an elevation in various cytokines and chemokines, including IL-6, IL-1β, and TGF-β, which are associated with Th17-polarization conditions (7) (Fig. 4A and B). While IL-23 levels remained undetectable, IL12/23p40 was increased by sCD14-stimulated monocytes (Figs. 4A and B and S6C).

In addition to cytokines, metabolites may play crucial roles in regulating T-cell differentiation and function (65). For instance, nitric oxide inhibits the transcriptional activity of RORγt and negatively influences Th17-cell differentiation (66). Therefore, to better understand what other factors secreted by monocytes may influence Th17 generation, we subjected the culture supernatant form sCD14-treated monocytes to metabolomic analysis. To account for the potential role of sex, we conducted our studies on monocytes isolated from males and age-matched females (*n* = 6/group). Metabolic pathway enrichment analysis revealed the activation of several pathways, including butanoate metabolism, arginine and proline metabolism, and alanine, aspartate, and glutamate metabolism (Fig. 4C). These changes in metabolic pathways were associated with a significant elevation in GABA and N (omega)-hydroxy-arginine levels only (Fig. 4C).

When we stratified the analysis for sex, only the butanoate metabolism pathway showed a consistent elevation in both sexes, resulting in increased levels of GABA in the culture supernatants of monocytes treated with sCD14 (Fig. 4D and E).

To investigate whether the elevated metabolites in the supernatant of sCD14-treated monocytes could drive T-cell polarization toward a Th17 phenotype, we supplemented T-cell culture media with GABA, hydroxyproline, and arginine. We found that GABA significantly increased IL-17A secretion by T cells but not hydroxyproline and arginine (Fig. 4F). This observation was further confirmed by qPCR (Fig. 4G). Our findings indicate that sCD14-induced GABA contributes to polarizing T cells toward a Th17 phenotype.

### Transcriptomic profiling of Th17 cells generated by sCD14-PBMC-Sup, GABA, and conventional Th17-polarizing condition

**Fig. 5.**
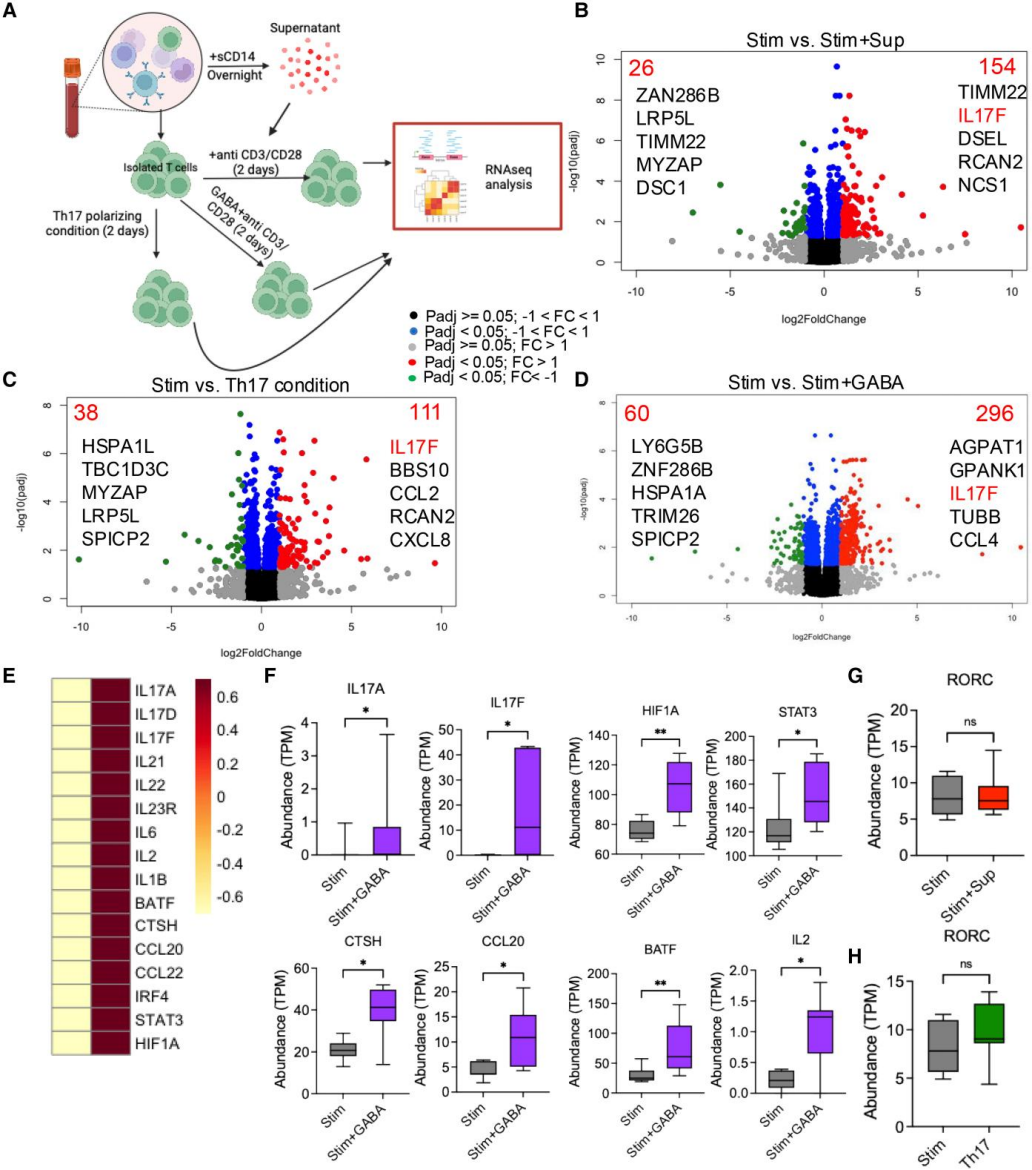
GABA promotes the generation of Th17 phenotype in T cells. A) Schematic figure illustrating the flow of our experimental design. Volcano plots showing differentially expressed genes in B) isolated T cells stimulated with anti-CD3/CD28, with and without treatment with the supernatant from sCD14-treated PBMCs, C) isolated T cells treated with Th17-polarizing conditions, and D) isolated T cells stimulated with anti-CD3/CD28 with and without GABA. E) Heat map and F) abundance (TMP) plots comparing differentially expressed genes associated with Th17 cells in isolated T cells stimulated with anti-CD3/CD28, with and without treatment with GABA. Abundance plots showing the expression of RORC gene in isolated T cells treated with G) the supernatant of sCD14-treated PBMCs or H) Th17-polarizing conditions. *P* values were calculated using two-tailed Mann–Whitney *U* test (F–H). *P < 0.05, **<0.01.

Given that different cues determine the fate of homeostatic versus harmful Th17 cells (67), we compared the transcriptional profile of Th17 cells generated with sCD14-PBMC-Sup to those generated using the conventional Th17-polarizing conditions. Similarly, we sought to characterize the transcriptional profile of total T cells treated with GABA. To do this, we isolated total T cells from HCs (*n* = 7) and cultured them under Th17-polarizing conditions, GABA, and sCD14-PBMC-Sup for 2 days (Fig. 5A). Bulk RNA-seq analysis revealed that 154 genes were significantly up-regulated and 26 genes were significantly down-regulated in total T cells treated with sCD14-PBMC-Sup, with IL-17F being among the five top up-regulated genes (Fig. 5B and Table S1). Similarly, we observed the up-regulation of 111 genes and down-regulation of 38 genes in T cells treated with Th17-polarizing condition, with IL-17F being the top up-regulated gene (Fig. 5C and Table S2). RNA-seq analysis of GABA-treated T cells showed up-regulation of 296 genes and down-regulation of 60 genes, with IL-17F among the top five up-regulated genes (Fig. 5D and Table S3). Further analysis of GABA-treated vs. control T cells revealed the up-regulation of genes associated with Th17-cell differentiation and function, particularly IL-17A, IL-17F, HIF-1A, STAT3, CTSH, CCL20, and BATF (Fig. 5E and F). However, the RORC gene was not up-regulated in GABA-treated T cells nor in T cells treated with sCD14 supernatant or Th17-polarizing conditions (Fig. 5G and H). This gene was supposedly to be down-regulated by 48 h posttreatment, when T cells were subjected to RNA-seq. In summary, our results support the role of GABA in the induction of Th17 phenotype, which is consistent with our ELISA and qPCR findings (Fig. 4F and G).

### sCD14 expands non-canonical type 2/3 ILCs

**Fig. 6.**
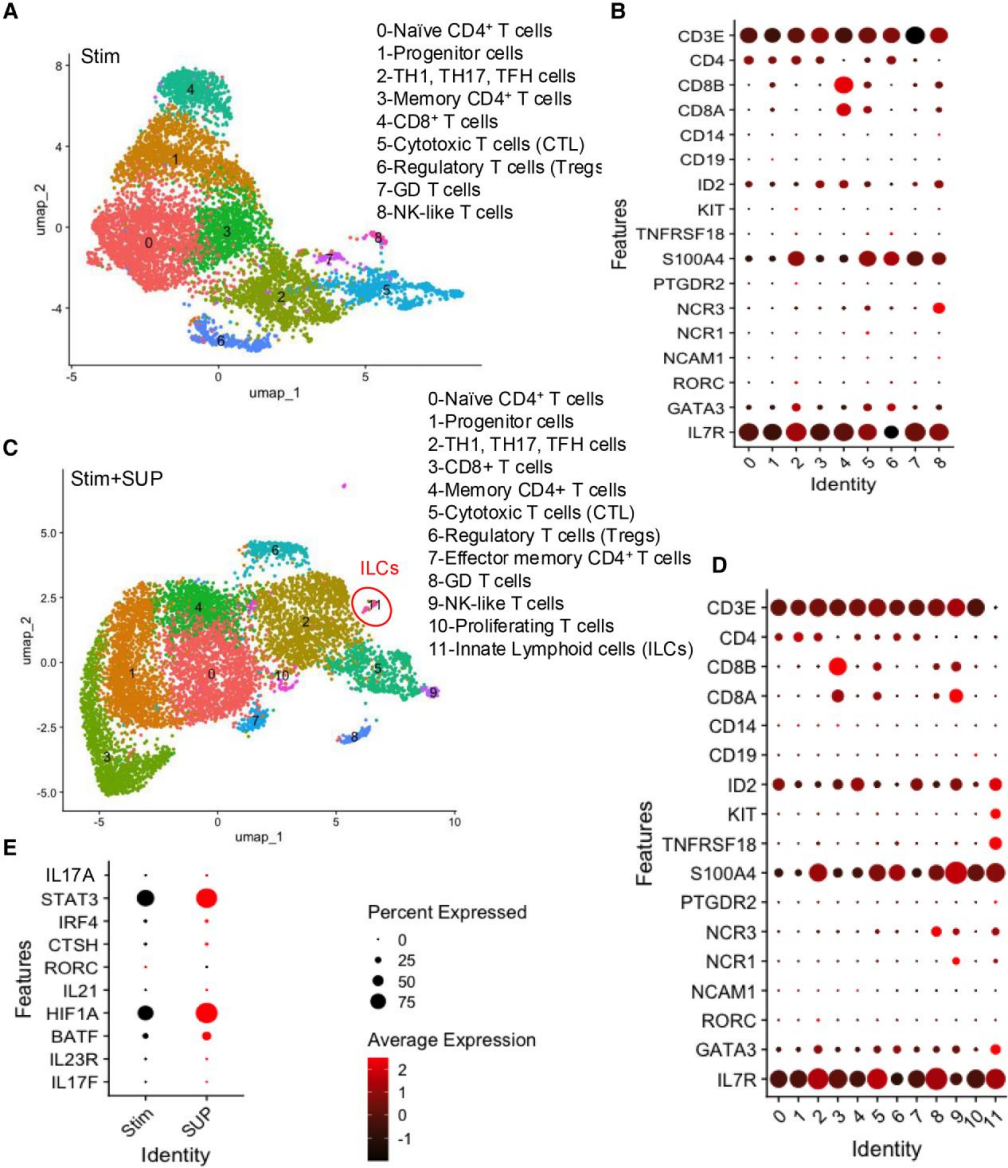
The supernatant of sCD14-treated PBMCs expands ILCs. A) UMAP plot and B) bubble plot showing different clusters of isolated T cells stimulated with anti-CD3/CD28. C) UMAP plot and D) bubble plot showing different clusters of T cells upon stimulation with anti-CD3/CD28 in the presence of the supernatant from sCD14-treated PBMCs. E) Density plot indicating the expression of IL17F gene in T cells cultured with supernatant from sCD14-treated PBMCs.

**Fig. 7.**
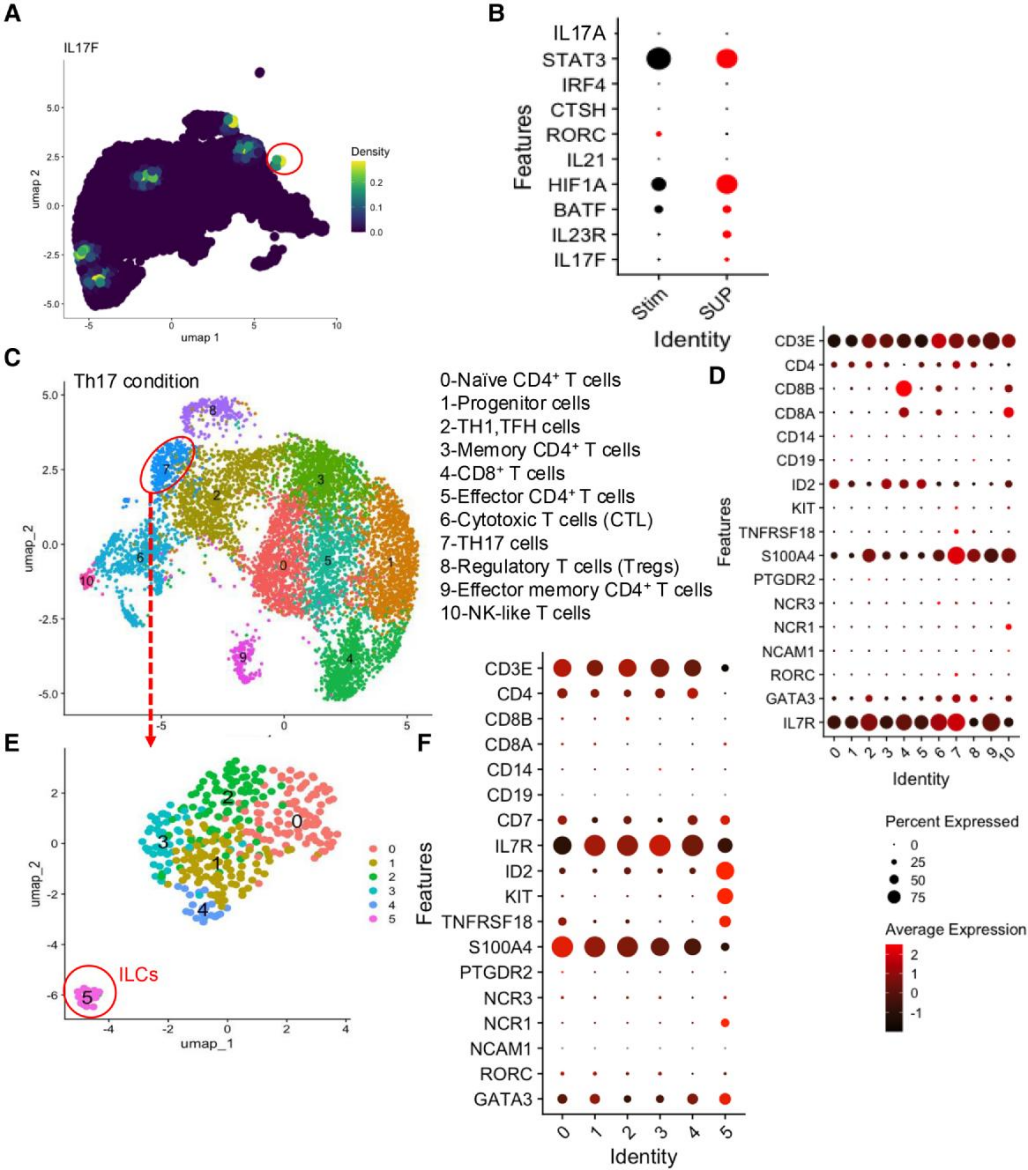
Th17-conditioning cytokines expands ILCs. A) UMAP plot and B) bubble plot of genes associated with Th17 cells in T cells stimulated in the absence or presence of the supernatant from sCD14-treated PBMCs. C) UMAP plot displaying different clusters of isolated T cells cultured under Th17-polarizing conditions. D) Density plots showing the expression of CD3E, CD4, CD8A, CD8B, CD14, and CD19 in isolated T cells cultured under Th17-polarizing conditions. E) UMAP plot and F) bubble plot showing subclusters within cluster 7 of isolated T cells cultured under Th17-polarizing conditions.

Given that IL-17 can be produced by various immune cells (7), we utilized scRNA-seq to gain deeper insights into the source of IL-17-secreting cells following treatment with sCD14. Isolated T cells from an HC were treated with autologous sCD14-PBMC-Sup. For comparison, we treated T cells from the same donor with either GABA or Th17-polarizing conditions for 2 days. scRNA-seq was performed after filtering out low-quality cells. We then applied unsupervised clustering via the Louvain algorithm (54) and visualized clustering for each condition using Uniform Manifold Approximation and Projection (UMAP). We identified nine clusters in the control condition and 12 clusters in the sCD14-PBMC-Sup condition, using the same clustering resolution (Figs. 6A–D and S7A and B). As expected, after merging the control and sCD14-PBMC-Sup conditions (Fig. S8A), we found that genes associated with the Th17 lineage, including IL17A, IL17F, STAT3, IRF4, BATF, CTSH, and HIF1A, were up-regulated in the sCD14-PBMC-Sup condition (Fig. 6E). Interestingly, and in contrast with our bulk RNA-seq data (Fig. 2D and E), the RORC gene was not up-regulated posttreatment and appears to be decreased. Notably, we identified a new cluster (cluster 11) in the sCD14-PBMC-Sup condition, which displayed an ILC gene signature (Fig. 6C and D). This cluster lacked the expression of traditional lineage markers like CD3E, CD4, CD8A, CD8B, CD14, and CD19 but expressed genes typically associated with ILCs, such as CD7 and IL17R (Figs. 6C and D and S8A). Further analysis revealed that this cluster exhibited high levels of IL-17F (Fig. 7A). This cluster showed the up-regulation of genes related to ILC3 function, including IL17A, IL17F, STAT3, HIF1A, BATF, and IL23R, in sCD14-PBMC-Sup-treated T cells (Fig. 7B).

**Fig. 8.**
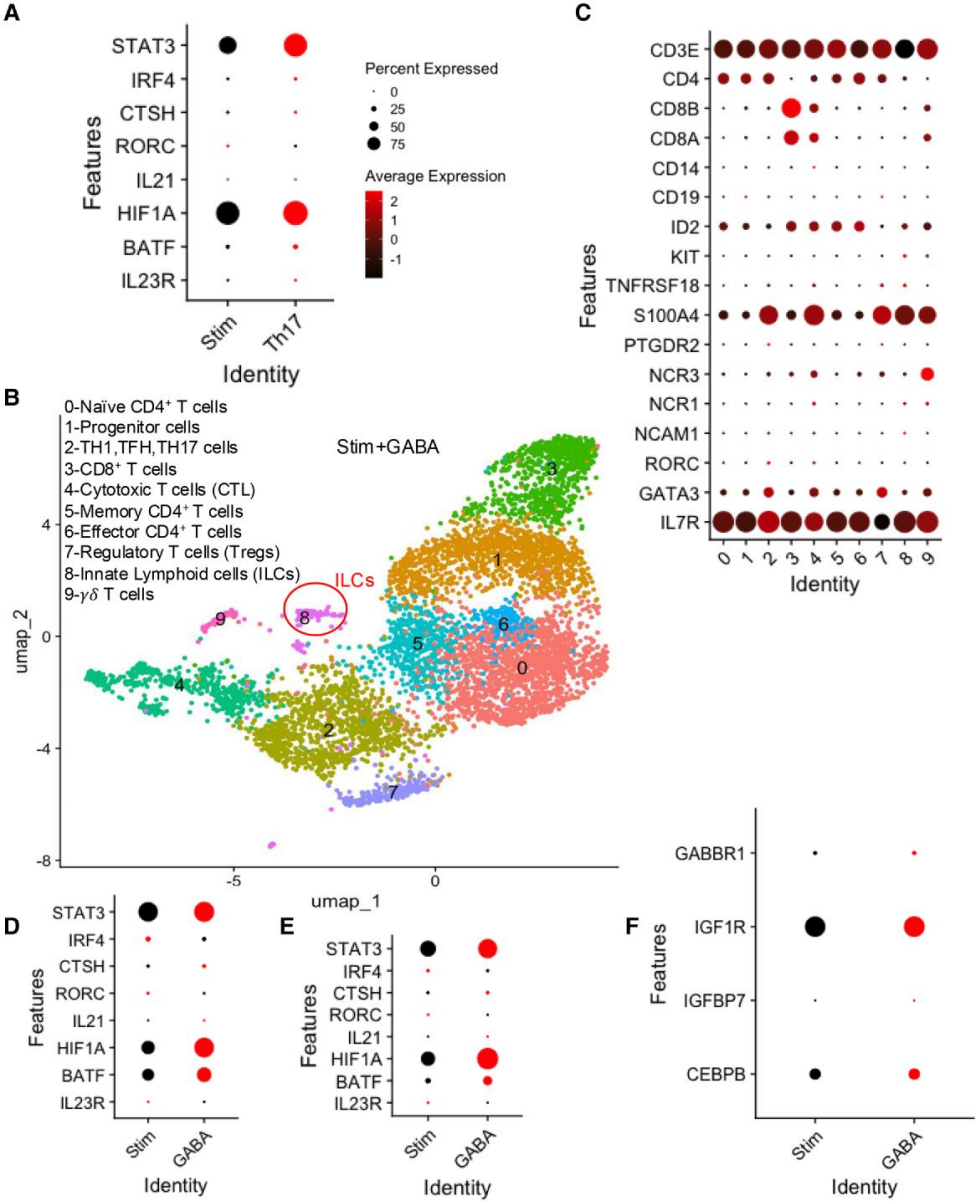
GABA promotes the expansion of ILCs in PBMCs. A) Bubble plot showing the expression of genes related to Th17 cells when total T cells are stimulated with anti-CD3/CD28 in the absence and presence of IL17 condition. B) UMAP plot and C) bubble plot showing different clusters of isolated T cells cultured with GABA and stimulated with anti-CD3/CD28. D) Bubble plot of differentially expressed genes associated with Th17 lineage in T cells stimulated with anti-CD3/CD28 in the absence or presence of GABA. E) Bubble plot of differentially expressed genes associated with Th17 lineage in cluster 8 of GABA-treated condition. F) Bubble plot of differentially expressed genes associated with GABA pathway in stimulated T cell alone or with GABA.

In the Th17-polarizing condition, we identified 11 clusters, with cluster 7 exhibiting a Th17-cell gene signature (Figs. 7C and D and S8B and C). Similar to the sCD14-PBMC-Sup condition, a small subset of cells (subcluster 5 within cluster 7) in the Th17-polarizing condition lacked lineage markers (CD3E, CD4, CD8A, CD8B, and CD14; Figs. 7E and F and S8D–F). After merging the control and Th17 conditions, further examination of subcluster 5 revealed up-regulation of genes associated with ILC3 function (Figs. 8A and S9A).

**Fig. 9.**
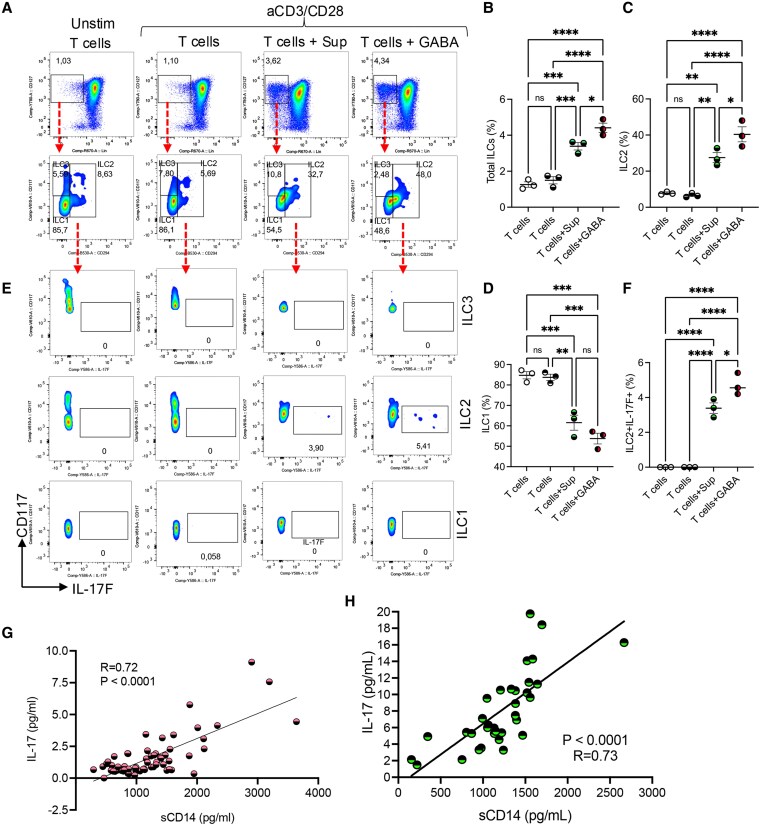
GABA and the supernatant from sCD14-treated PBMCs promote the expansion of IL-17 secreting ILC2s. A) Representative flow cytometry plots, B) cumulative data of total ILCs, C) ILC2, and D) ILC1 subsets in T cells treated under indicated conditions for 48 h. E) Representative flow plots and F) cumulative data of percentages of ILC2 expressing IL-17F in T cells treated under indicated conditions for 42 h plus 6 h with a golgi blocker. G) The correlation of sCD14 with IL-17 in the plasma of SARS-CoV-2-infected individuals with moderate and severe disease. H) The correlation of sCD14 with IL-17 in the plasma of LC patients with ME/CFS. ANOVA (B–D, F) and the Spearmen correlation analysis (G, H). *P < 0.05, **<0.01, ***<0.001, ****<0.0001. Not significant (ns). Immune cell lineages (Lin).

In the GABA-treated condition, 10 clusters were also present, with cluster 8 showing an ILC gene signature (Figs. 8B and C and S9B). Additionally, after merging with the control condition (Fig. S9C), we observed up-regulation of genes related to the Th17-cell lineage in GABA-treated T cells as well (Fig. 8D). Furthermore, Th17-cell lineage genes were up-regulated in cluster 8, suggesting that this cluster comprises ILC3-like cells (Fig. 8E). To confirm our ssRNA-seq observations, isolated T cells were cultured for 2 days either unstimulated or stimulated with anti-CD3/CD28 in the presence of GABA or sCD14-PBMC-Sup derived from autologous PBMCs. We found that both sCD14-PBMC-Sup and GABA significantly increased the proportion of total ILCs (Fig. 9A and B). Further analysis revealed a significant expansion of ILC2s accompanied by a reduction in the proportion of ILC1s, with no substantial change in the frequency of ILC3s under both conditions (Fig. 9A, C, and D). Additionally, we found that IL-17F expression originated exclusively from the ILC2 subset in our system (Fig. 9E and F). Notably, a recent study reported that GABA suppresses the TF C/EBP-β by inhibiting Igfbp7 in an autocrine manner via the Igf1R receptor (68). This suppression subsequently reduces ILC3 activity and IL-17 production. However, our observations indicate that GABA up-regulates the expression of C/EBP-β, Igf1R, Igfbp7, and GABR1 in ILCs (Fig. 8F). These findings suggest that GABA promotes IL-17 production through the expansion of ILC2s in vitro.

### The plasma sCD14 levels are positively correlated with IL-17A

Increased plasma levels of sCD14 in rheumatoid arthritis, systemic lupus erythematosus, and cardiovascular diseases are associated with disease activity (69–71). Similarly, sCD14 is elevated in HIV infection and is predictive of morbidity and mortality (72). Particularly, increased plasma sCD14, intestinal fatty acid–binding protein, and lipopoly saccharide (LPS) are associated with microbial translocation in HIV (73). Additionally, the elevation of sCD14 in acute COVID-19 disease and long COVID (LC) patients has been reported (40, 41). Therefore, we decided to determine whether elevated levels of sCD14 are linked to increased IL-17A in these patients. Intriguingly, we found a strong positive correlation between the plasma sCD14 levels and IL-17A in both acute COVID-19 and LC patients, respectively (Fig. 9G and H). These observations suggest that elevated sCD14 may, at least in part, explain the increased frequency of IL-17-producing cells (74) and elevated plasma IL-17A levels in acute COVID-19 (41 ) and LC patients with ME/CFS (46, 75, 76).

## Discussion

In this study, we demonstrated that sCD14 has a pivotal role in promoting the differentiation and expansion of Th17 cells, both from naive and pre-existing Th17-cell subsets in PBMCs. Through comprehensive analyses, we found that sCD14 enhances Th17-associated cytokine expression/production (e.g. IL-17A, IL-17F, IL-21, and IL-22), promotes the up-regulation of key TFs such as STAT3, RORC, and increases the secretion of IL-17A, a hallmark of Th17 cells. Importantly, our results also reveal that sCD14 acts indirectly through autologous sCD14-PBMC-Sup, suggesting a complex signaling pathway driving Th17 polarization.

Our findings that sCD14 induces Th17 polarization are in line with previous studies suggesting that sCD14 can influence T-cell effector functions (44). Upon culturing T cells with sCD14-PBMC-Sup, we observed up-regulation of several Th17-associated genes, including IL-17A, IL-17F, IL-21, and IL-22, alongside the activation of critical TFs, notably STAT3 and RORC (5, 8). These results suggest that sCD14 facilitates the differentiation of naive T cells into a Th17 phenotype or expands pre-existing Th17 subsets. The significant up-regulation of IL-17A expression and secretion confirmed this functional outcome.

Interestingly, our investigation into the cytokine profiles of sCD14-PBMC-Sup-treated T cells reveals a distinct secretion pattern that was enriched for pro-inflammatory cytokines like MCP-1, MCP-4, IP-10, MDS, and TARC, suggesting a robust skewing environment. While monocytes and macrophages are generally considered as the primary source of these chemokines, they can be secreted by activated T cells (77–80). Similarly, the expression of Tie2 in activated T cells has been reported (81). Notably, higher levels of IL-8 in the conventional Th17-polarizing condition points to the activation of a subset of IL-8-producing T cells observed under these conditions (82). This was contrasted by the lower secretion of Th1-related cytokines such as IFN-γ, reinforcing the Th17-promoting effect of sCD14. Importantly, the fact that both naive CD4 and CD8 T cells responded similarly to sCD14-PBMC-Sup in comparison to the conventional Th17-polarizing conditions, points to a broader capacity of sCD14 to polarize multiple T-cell subsets toward a Th17 phenotype and shape T-cell-mediated immunity.

While sCD14-activated monocytes secrete a wide range of cytokines and chemokines, only a few of them, including IL-6, IL-1, and TGF-β, may promote Th17 differentiation.

An intriguing aspect of our study is the identification of GABA, a metabolite elevated in monocytes treated with sCD14, as a potential contributor to Th17 polarization. Our metabolic analysis of sCD14-treated monocytes revealed a distinct activation of metabolic pathways, including the butanoate metabolism pathway, leading to increased GABA secretion. The capability of monocytes to secrete GABA is supported in macrophages, monocytes, and DCs in both mice and humans (83, 84). Furthermore, the vesicular inhibitory amino acid transporter, which transports GABA into synaptic vesicles, has been detected in human monocytes (84). When supplemented into T-cell cultures, GABA enhanced IL-17A secretion, a result confirmed by qPCR. This suggests that GABA may serve as a novel signaling molecule that amplifies the Th17 response, supporting the hypothesis that metabolites can play critical roles in T-cell differentiation (85). GABA exerts its effects via two receptors, GABA-A and GABA-BB (86). However, multiple GABA receptor subunits have been identified in various immune cells (83, 85). Although it is evident that T cells can express GABA-A subunits, the factors determining specific receptor subtypes in T cells remain unclear. While sCD14-induced GABA production contributes to Th17 polarization, further studies are required to unravel the exact mechanisms by which GABA interacts with T cells to influence this process.

A particularly interesting finding in this study was the identification of ILC2/3-like cells in T-cell cultures exposed to sCD14-PBMC-Sup, as revealed by scRNA-seq. These cells, which lacked traditional T-cell lineage markers (CD3, CD4, and CD8), expressed genes typically associated with ILC3 function, including IL-17F, STAT3, and BATF. Since isolated T cells were used for our scRNA-seq analysis, the emergence of ILCs suggests the presence of ILC precursors within the T-cell subset. This likely occurred due to the negative T-cell isolation technique, which may have allowed ILCs—lacking lineage markers—to be included in the isolated T cell. This suggests that sCD14 not only influences conventional T cells but may also expand or induce a subset of ILCs that contribute to the Th17 response (87, 88). The identification of ILC2/3-like cells in both sCD14-PBMC-Sup and Th17-polarizing conditions further emphasizes the complex interplay between different immune cell populations in driving Th17-mediated immunity. Although our scRNA-seq data support the expansion of ILC3 subsets, our flow cytometry findings indicate the expansion of IL-17-secreting ILC2 subsets in response to both sCD14-PBMC-Sup and GABA. These results support the concept that the tissue microenvironment can shape ILC plasticity (22).

While sCD14 appears to have a significant role in Th17 polarization, it also exerts immunosuppressive effects on T cells (44). Our data showing the inhibition of cytokine production in T cells, alongside reduced activation marker expression, indicates that sCD14 impairs conventional T-cell activation and proliferation. Notably, this suppression was observed only when T cells were cultured in the presence of sCD14-PBMC-Sup, suggesting that the immunosuppressive effects of sCD14 are mediated indirectly through its interactions with other PBMC components, particularly monocytes. Given the significant up-regulation of CTLA-4 in T cells, it is plausible that sCD14-PBMC-Sup induces T-cell suppression through enhanced CTLA-4 expression as an alternative inhibitory mechanism (89). However, our experiments did not reveal any direct effects of sCD14 on T cells, supporting the idea that its immunosuppressive actions are likely mediated by soluble factors released by monocytes in response to sCD14. Collectively, our findings suggest that sCD14 skews T cells toward a Th17 phenotype, at least in part via activation of the GABA-mTOR signaling pathway (90). Additionally, an enhanced IL-17 expression in the intestinal tissues following enterotoxigenic *Escherichia coli* infection by GABA has been reported (20). Nevertheless, the immunomodulatory properties of GABA remain controversial. For instance, GABA signaling has been shown to reduce inflammation in EAE (83) and rheumatoid arthritis models (91). Moreover, a recent study reported that GABA suppresses ILC3 activity and IL-17 production in the gut via inhibition of C/EBP-β-Igfbp7 pathway, a finding that sharply contrasts with ours (68). In our study, GABA promotes this pathway, as evidenced by the up-regulation of *CEBPB*, *IGFbp7*, and *GABAR1* gene expression. This discrepancy may be due to differences between the in vivo model used in that study and our in vitro human system.

Our data suggest that sCD14 effects are primarily mediated via TLRs, particularly TLR4. Treatment of PBMCs with sCD14 elicited a classical TLR4-dependent MyD88/NF-κB program in monocytes (31), evidenced by increased TNF-α, IL-1α, IL-8, CCL2 (MCP-1), and CCL4 (MIP-1β), accompanied by TRIF/STAT1-linked iNOS activation, as indicated by accumulation of N(ω)-hydroxy-L-arginine (92). Concomitant changes in GABA and 4-hydroxyproline are consistent with GABA-shunt engagement and matrix/remodeling by-products downstream of monocyte activation. We also observed elevated TIE-2 levels, likely reflecting expansion of Tie2⁺ monocytes and/or metalloprotease-mediated shedding (93). Notably, the cytokine/metabolite profile aligns with TLR4 priming (Signal-1) rather than full inflammasome activation, as IL-1α increased without evidence for caspase-1 processing or mature IL-1β production. Future studies using pharmacologic inhibition of TLR4, together with NLRP3 blockade during a second-signal challenge, will be valuable to directly validate these pathways.

## Conclusion

This study highlights the complex role of sCD14 in regulating T-cell responses, specifically its ability to polarize T cells toward a Th17 phenotype while simultaneously suppressing T-cell effector functions. The identification of GABA and ILC2/3-like cells as potential contributors to Th17 differentiation provides new insights into the multifaceted nature of immune regulation by sCD14. Further studies are needed to clarify the mechanisms by which sCD14 mediates both Th17 expansion and T-cell suppression, as well as the functional consequences of GABA-mediated signaling in immune responses. Additionally, further studies are required to validate and assess the effects of sCD14-PBMC-Sup and GABA on sorted ILCs.

The findings presented here open up several important avenues for future research, particularly in the context of autoimmune diseases, where Th17 cells play a critical role. Understanding how sCD14 modulates immune responses could provide new therapeutic strategies for manipulating Th17-driven immunity in a variety of disease settings. Thus, targeting sCD14 may hold therapeutic potential in conditions where elevated levels of sCD14 promote a Th17 phenotype.

## Supplementary Material

pgaf406_Supplementary_Data

## Data Availability

Generated data are publicly available from the SRA portal of NCBI under the accession numbers GSE277708, GSE277711, SAMN47343883, SAMN47343884, SAMN47343885, and SAMN47343886.
