# Correction to: Soluble CD14 promotes Th17 expansion and differentiation through gamma-aminobutyric acid and expands non-canonical innate lymphoid cells

**DOI:** 10.1093/pnasnexus/pgag147

**Published:** 2026-05-12

This is a correction to: Shima Shahbaz, Amirhossein Rahmati, Hussain Syed, Shokrollah Elahi, Soluble CD14 promotes Th17 expansion and differentiation through gamma-aminobutyric acid and expands non-canonical innate lymphoid cells, *PNAS Nexus*, Volume 5, Issue 1, January 2026, pgaf406, https://doi.org/10.1093/pnasnexus/pgaf406

A change has been made to the title of this paper and the sub-heading proceeding figure 5.

The new title is as follows:

**Soluble CD14 promotes Th17 expansion and differentiation through gamma-aminobutyric acid and expands non-canonical innate lymphoid cells**

The new sub-heading is as follows:

**sCD14 expands non-canonical type 2/3 ILCs**

