# Supplementary material for: Soluble CD14 promotes Th17 expansion and differentiation through gamma-aminobutyric acid and expands non-canonical innate lymphoid cells
Source: PNAS Nexus. 2025 Dec 29;5(1):pgaf406. doi: 10.1093/pnasnexus/pgaf406 (PMC12781096; doi:10.1093/pnasnexus/pgaf406)
Supplement: pgaf406_Supplementary_Data [file pgaf406_supplementary_data.zip › PNASNEXUS-PNASNEXUS-2025-00909RR-s01.pdf]

Soluble CD14 promotes Th17 expansion and differentiation through GABA and expands infidel ILCs

Shima Shahbaz[1], Amirhossein Rahmati[1], Hussain Syed[2], and Shokrollah Elahi[1, 3-5*]

[1]Mike Petryk School of Dentistry, Division of Foundational Sciences, [2]Department of Medicine, Division of Gastroenterology, [3]Li Ka Shing Institute of Virology, [4]Women and Children Health Research Institute, [5]Cancer Research Institute of Northern Alberta, [6]Glycomics Institute of Alberta, Faculty of Medicine and Dentistry, University of Alberta, Edmonton, T6G 2E1, AB, Canada.

*Corresponding author

Shokrollah Elahi, MD, PhD

elahi@ualberta.ca

ORCID# 0000-0002-7215-2009

Supplemental Figures

Fig. S1. **A**) Representative flow cytometry plot showing the purity of total T cells, **B**) the purity of CD4 T cells and the naïve subset, **C**) the purity of isolated naive CD8 T cells, and **D**) the purity of monocytes pre-and post-enrichment. **E**) Representative flow cytometry plots and cumulative data of percentages of CD8 and CD4 T cells expressing IFN-γ when total PBMCs were stimulated with anti-CD3/CD28 and treated with and without sCD14. *P* values were calculated using two tailed Mann–Whitney U test (E); ** < 0.01. Each dot represents sample from a study subject.

Fig. S2. **A**) Representative flow cytometry plots of CD25 and CD69 expression in CD4, and **B**) CD8 T cells in the absence of stimulation (non-stim), stimulated (stim) with anti-CD3/CD28 in the presence and absence of sCD14. **C**) Cumulative data of percentages of CD4+CD25+ T cells, **D**) CD4+CD69+ T

cells, **E**) CD8+CD25+ T cells, and **F**) CD8+CD69+ T cells either non-stim, stim without or with sCD14 overnight. **G**) Representative flow cytometry plots, and **H**) cumulative data of the percentages of apoptotic and dead CD4 and CD8 T cells in indicated conditions. **I**) Representative flow cytometry plots of the gating strategy for the frequency of M-MDSCs in the absence and presence of sCD14. *P* values were calculated using two tailed Mann–Whitney U test (**I**) and Paired comparisons were analyzed using the Wilcoxon matched-pairs signed-rank test (C-F, H). ** < 0.01, *** < 0.001. Each dot represents sample from a study subject. Fluorescence minus one (FMO) and dimethyl sulfoxide (DMSO).

Fig. S3. **A**) Representative flow cytometry plots of TNF-α and IFN-γ expression in isolated CD4 and CD8 T cells after treatment with anti-CD3/CD28 in the presence and absence of sCD14. **B**) Cumulative data of TNF-α and IFN-γ expression in CD4, and **C**) CD8 T cells stimulated with anti-CD3/CD28 sCD14 vs non-treated isolated CD4 and (**C**) CD8 T cells. **D**) Representative flow cytometry plots of TNF-α production from isolated T cells and CD4 and CD8 T cells from total PBMCs stimulated with anti-CD3/CD28 in the presence and absence of sCD14-PBMC-Sup. **E)** Cumulative data of TNF-α production from isolated CD4 T cells**. F)** Abundance (TPM) of various genes in stimulated cultures compared to those treated with culture supernatant (Sup). *P* values were calculated using two tailed Mann–Whitney U test (**F**) and Paired comparisons were analyzed using the Wilcoxon matched-pairs signed-rank test (B-E). ** < 0.01, *** < 0.001, **** < 0.0001. Each dot represents sample from a human study subject

Fig. S4. Plots showing the log fold change and -log10 (*p*-value) of the genes related to **A)** RORC, **B)** STAT3, and **C)** HIF1A pathways. Box plots showing the gene expression of various genes associated with **D)** Th1, **E)** Th2 cells, and **F)** Tregs in isolated T cells, with or without treatment with sCD14-PBMC-Sup. **G)** Cumulative data of TPM of TNF-α and **H)** IFN-γ genes in T cells treated with sCD14-PBMC-Sup. *P* values were calculated using two tailed Mann–Whitney U test (**D-H**).

Fig. S5. A) Fold change expression of CTLA-4 in T cells stimulated with culture supernatant from untreated autologous PBMCs with sCD14 or those treated with culture supernatants from PBMCs treated with sCD14 as measured by qPCR. **B**) Cumulative data of Th17-related cytokines measured in culture supernatants of total T cells stimulated with anti-CD3/CD28 (stim) alone or plus IL-17 polarizing condition. **C**) Detectable cytokines and chemokines in culture supernatants from culture supernatants of PBMCs without treatment with sCD14 (-) or treated with sCD14 (+) overnight and measured by multiplex ELISA (Meso Scale Discovery platform). *P* values were calculated using two tailed Mann–Whitney U test (A,C) and Paired comparisons were analyzed using the Wilcoxon matched-pairs signed-rank test (B). * < 0.05, ** < 0.01, *** < 0.001, **** < 0.0001. Each dot represents sample from a human study subject. Data are from 8 study subjects (C).

Fig. S6. A) Cumulative data of Th17-related cytokines measured in culture supernatants of total T cells stimulated with anti-CD3/CD28 plus autologous culture supernatants untreated with sCD14 compared to T cells stimulated with anti-CD3/CD28 plus autologous culture supernatants treated with sCD14. **B**) Representative and cumulative data showing percentages of proliferating IL-17F+ CD4 and CD8 T cells in indicated conditions when analyzed 96 hours later. **C**) Cumulative data of TGF-β1 levels detected in culture supernatants of monocytes treated without or with sCD14. *P* values were calculated using the Wilcoxon matched-pairs signed-rank test (A-C). * < 0.05, ** < 0.01. Each dot represents sample from a human study subject.

Fig. S7. Heatmap showing the row-scaled expression of the top five highest differentially expressed genes (DEGs) (Bonferroni-corrected P values < 0.05; Student's t-test) per cluster of isolated T cells **A)** stimulated with anti-CD3/CD28 and **B)** cultured with sCD14-PBMC-Sup.

Fig. S8. A) UMAP plot of merged control and sCD14-PBMC-Sup conditions. **B**) Heatmap showing the row-scaled expression of the top five highest DEGs (Bonferroni-corrected P values < 0.05; Student's t-test) per cluster of isolated T cells culture under Th17-polarizing condition. **C)** Density plot showing the gene expression of RORC in T cells cultured under Th17-polarizing conditions. **D-F)** Feature plots

showing the expression of CD7, ID2, and IL7R in different subclusters of Cluster 7 from T cells cultured under Th17-polarizing conditions.

Fig. S9. A) UMAP plot of merged control and Th17 polarizing conditions. **B)** Heatmap showing the row-scaled expression of the top five highest DEGs (Bonferroni-corrected P values < 0.05; Student's t-test) per cluster of isolated T cells cultured with GABA**. C)** UMAP plot of merged control and GABA conditions.

Table S1. The complete list of up-and-downregulated genes related to Fig. 5B.

Table S2. The complete list of up-and-downregulated genes related to Fig. 5C.

Table S3. The complete list of up-and-downregulated genes related to Fig. 5D.

S Fig. 1

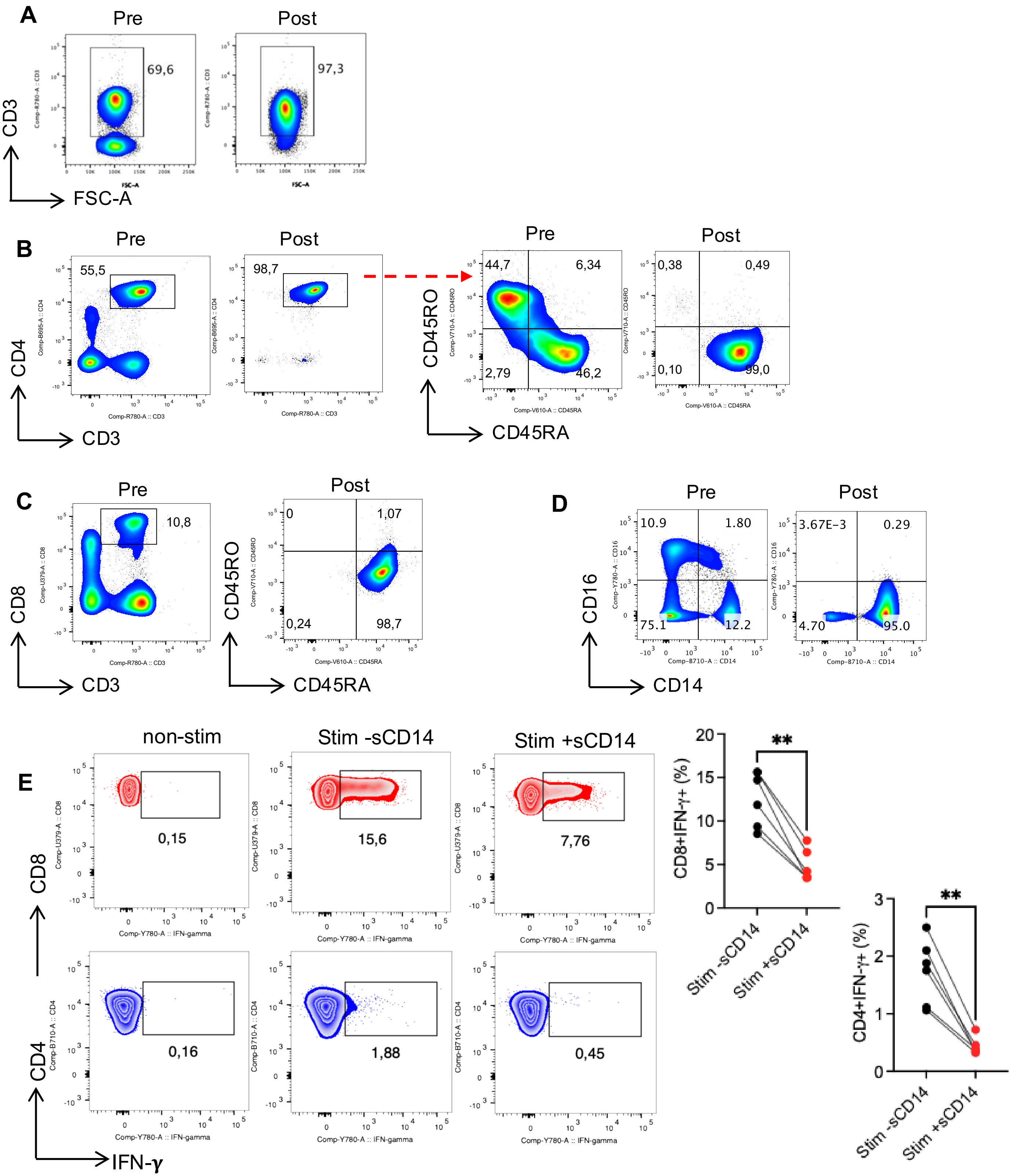

A
Pre
Post
69,6
97,3
CD3
FSC-A
B
Pre
Post
55,5
98,7
44,7
6,34
2,79
46,2
0,38
0,49
0,10
99,0
CD4
CD3
CD45RO
CD45RA
C
Pre
Post
10,8
0
1,07
0,24
98,7
CD8
CD3
CD45RO
CD45RA
D
Pre
Post
10.9
1.80
75.1
12.2
3.67E-3
0.29
4.70
95.0
CD16
CD14
E
non-stim
Stim -sCD14
Stim +sCD14
0,15
15,6
7,76
0,16
1,88
0,45
CD8
CD4
IFN-γ
CD8+IFN-γ+ (%)
CD4+IFN-γ+ (%)
**
Stim -sCD14
Stim +sCD14

S Fig. 2

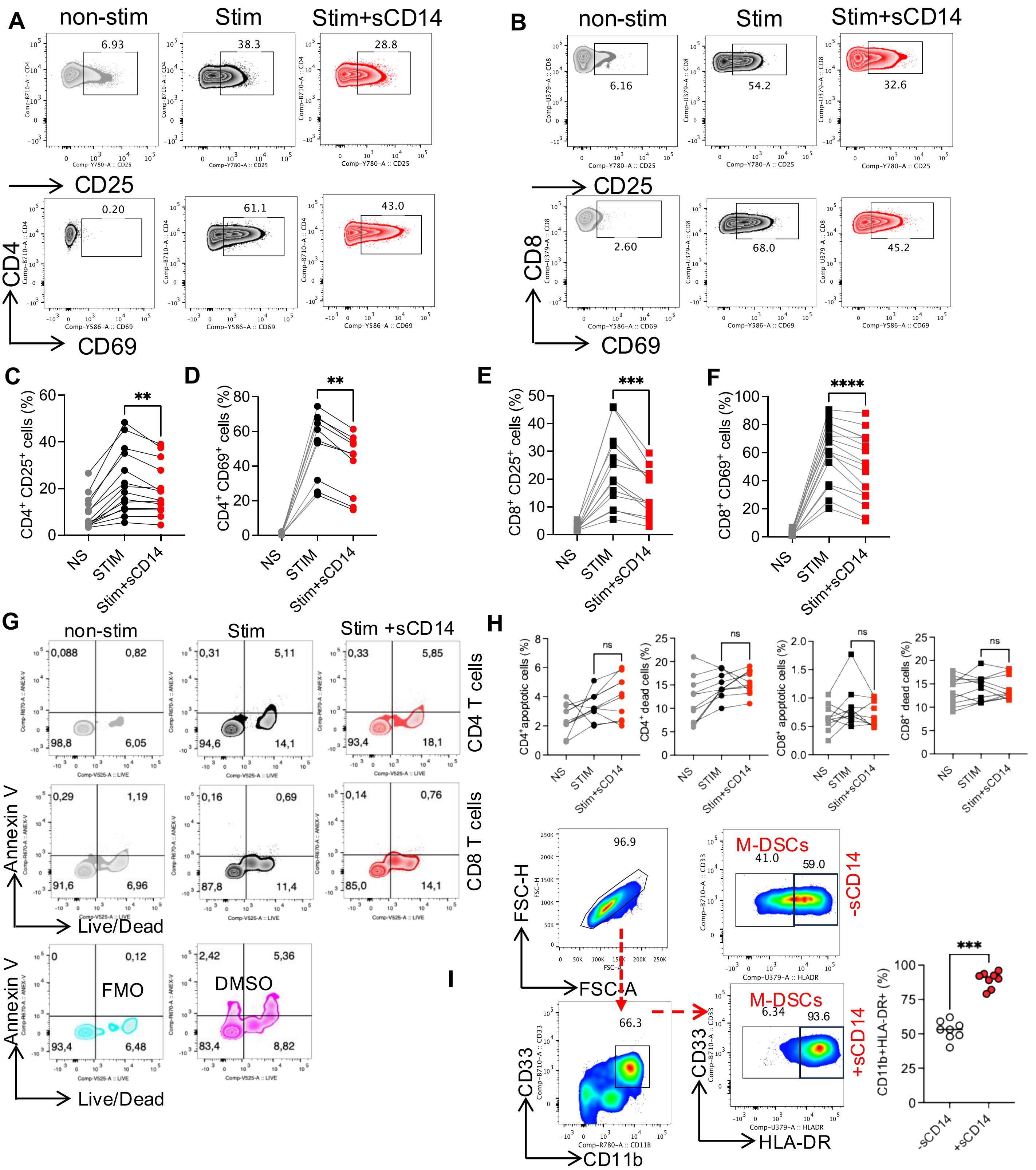
A
non-stim
Stim
Stim+sCD14
6.93
38.3
28.8
CD25
0.20
61.1
43.0
CD4
CD69
B
non-stim
Stim
Stim+sCD14
6.16
54.2
32.6
CD25
2.60
68.0
45.2
CD8
CD69
C
CD4+ CD25+ cells (%)
**
NS
STIM
Stim+sCD14
D
CD4+ CD69+ cells (%)
**
E
CD8+ CD25+ cells (%)
***
F
CD8+ CD69+ cells (%)
****
G
non-stim
Stim
Stim +sCD14
0,088
0,82
98,8
6,05
0,31
5,11
94,6
14,1
0,33
5,85
93,4
18,1
CD4 T cells
0,29
1,19
91,6
6,96
0,16
0,69
87,8
11,4
0,14
0,76
85,0
14,1
CD8 T cells
Annexin V
Live/Dead
0
0,12
FMO
93,4
6,48
2,42
5,36
DMSO
83,4
8,82
H
CD4+apoptotic cells (%)
CD4+ dead cells (%)
CD8+ apoptotic cells (%)
CD8+ dead cells (%)
ns
I
FSC-H
FSC-A
96.9
CD33
CD11b
66.3
M-DSCs
41.0
59.0
-sCD14
M-DSCs
6.34
93.6
+sCD14
HLA-DR
CD11b+HLA-DR+ (%)
***
-sCD14
+sCD14

S Fig. 3

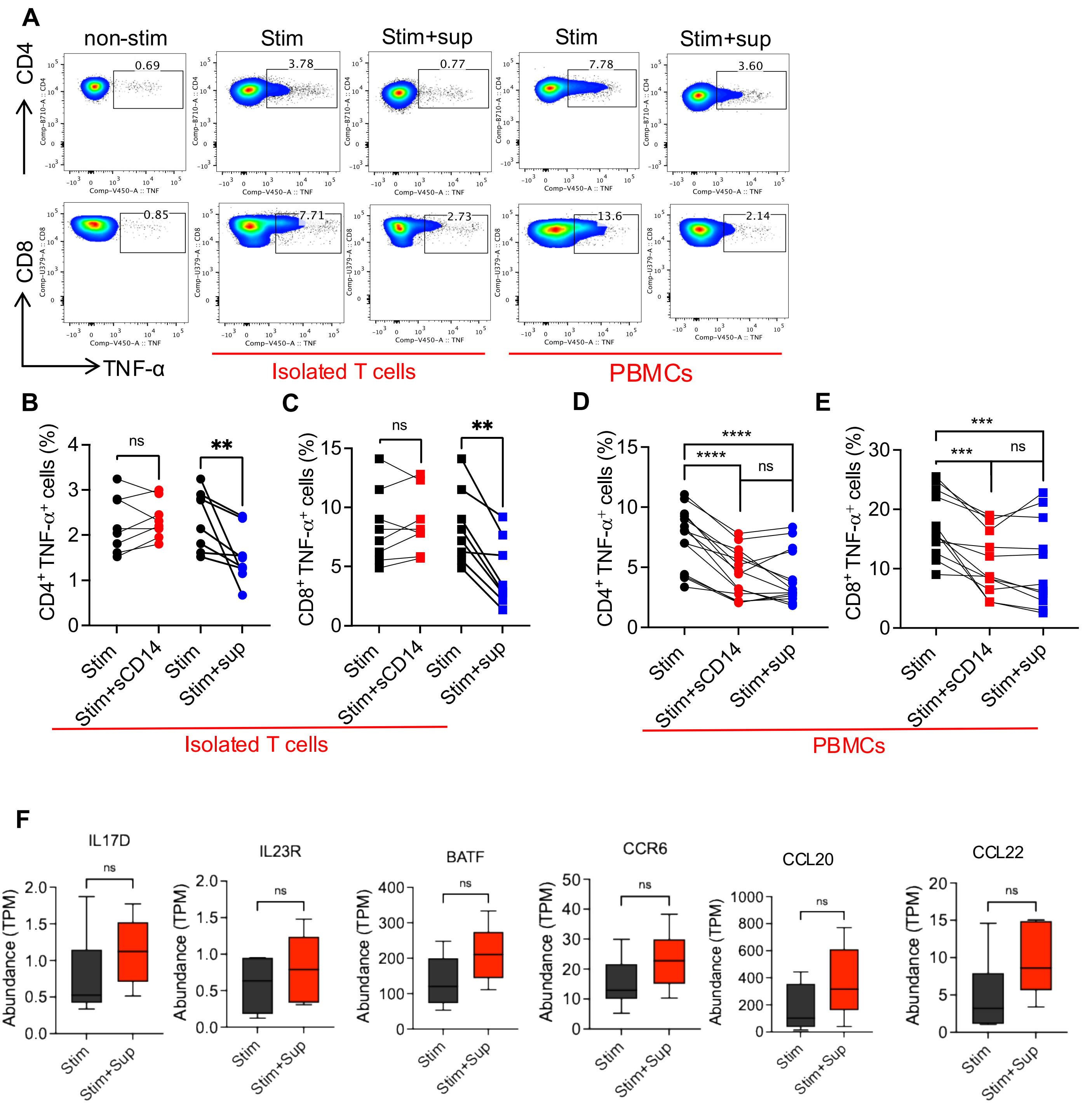
A
non-stim
Stim
Stim+sup
Stim
Stim+sup
CD4
CD8
TNF-α
0.69
3.78
0.77
7.78
3.60
0.85
7.71
2.73
13.6
2.14
Isolated T cells
PBMCs
B
C
D
E
CD4+ TNF-α+ cells (%)
CD8+ TNF-α+ cells (%)
Stim
Stim+sCD14
Stim+sup
ns
**
****
***
Isolated T cells
PBMCs
F
IL17D
IL23R
BATF
CCR6
CCL20
CCL22
Abundance (TPM)
Stim
Stim+Sup
ns

S Fig. 4

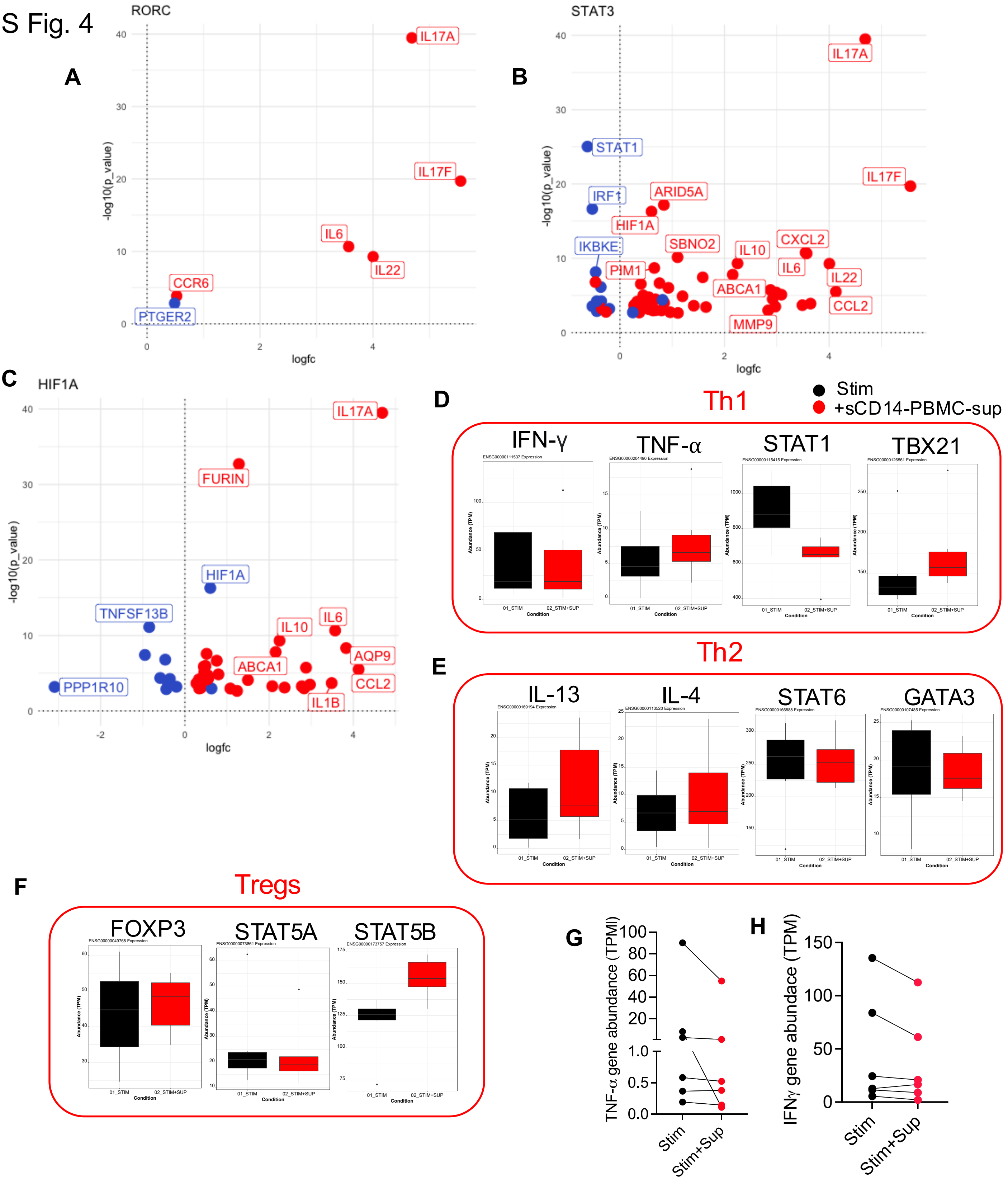

A
RORC
IL17A
IL17F
IL6
IL22
CCR6
PTGER2
-log10(p_value)
logfc
B
STAT3
IL17A
STAT1
IRF1
ARID5A
IL17F
HIF1A
IKBKE
SBNO2
IL10
CXCL2
PIM1
IL6
IL22
ABCA1
CCL2
MMP9
-log10(p_value)
logfc
C
HIF1A
IL17A
FURIN
HIF1A
TNFSF13B
IL10
IL6
AQP9
ABCA1
CCL2
PPP1R10
IL1B
-log10(p_value)
logfc
D
Th1
Stim
+sCD14-PBMC-sup
IFN-γ
TNF-α
STAT1
TBX21
Abundance (TPM)
01_STIM
02_STIM+SUP
Condition
E
Th2
IL-13
IL-4
STAT6
GATA3
Abundance (TPM)
01_STIM
02_STIM+SUP
Condition
F
Tregs
FOXP3
STAT5A
STAT5B
Abundance (TPM)
01_STIM
02_STIM+SUP
Condition
G
TNF-α gene abundance (TPM)
Stim
Stim+Sup
H
IFNγ gene abundace (TPM)
Stim
Stim+Sup

S Fig. 5

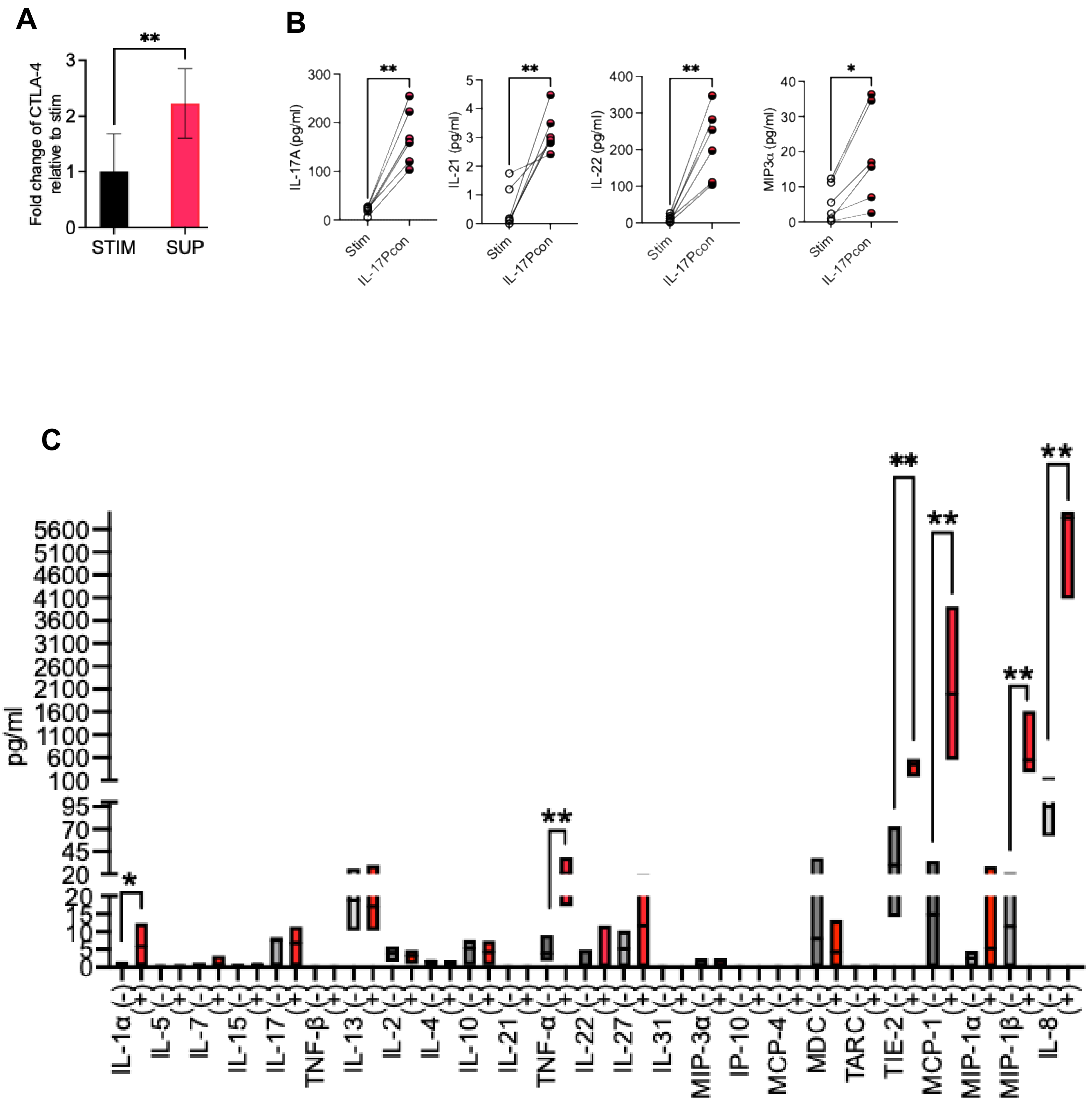

Culture supernatant without sCD14 (-)
Culture supernatant with sCD14 (+)

S Fig. 6

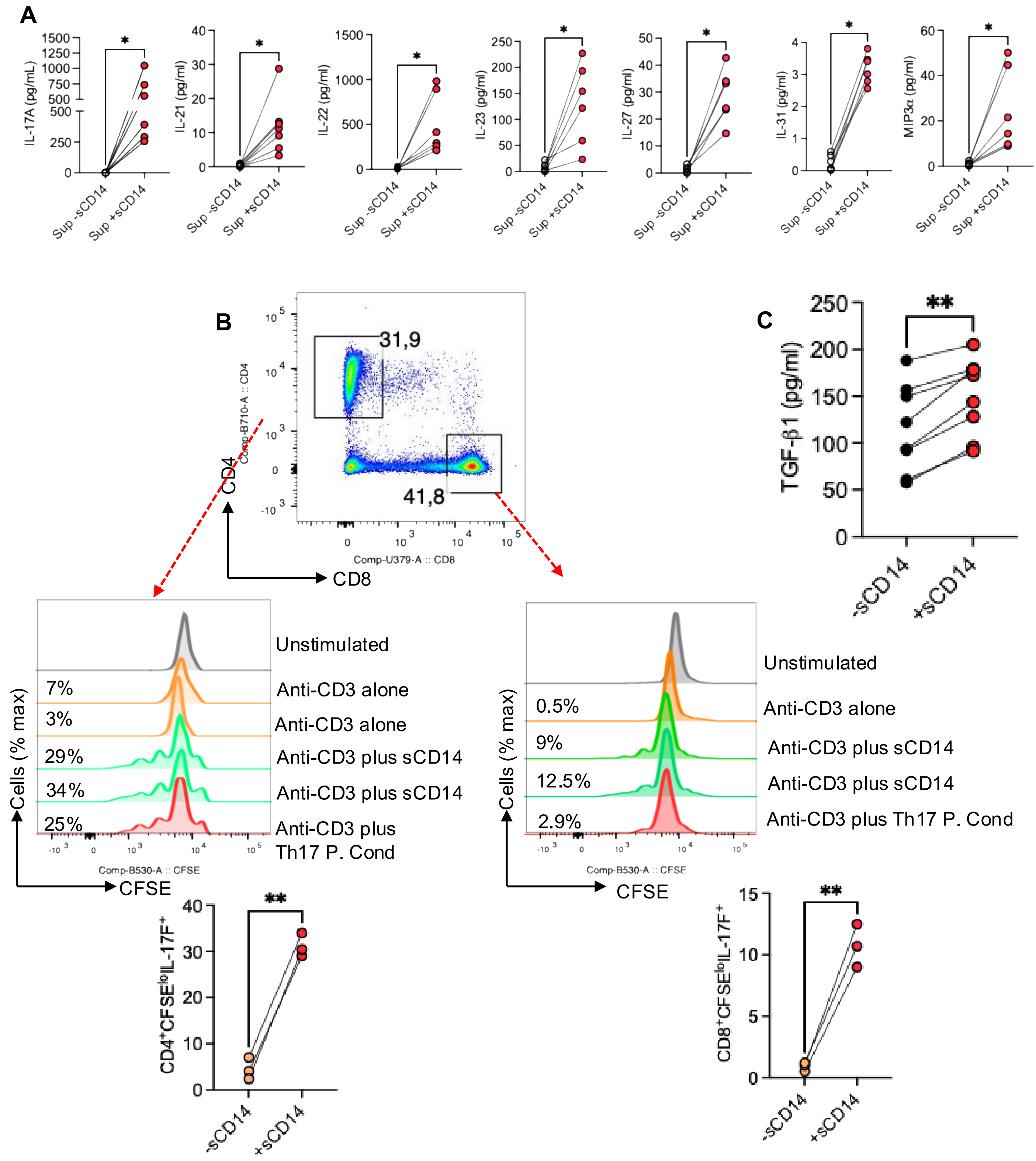
A
IL-17A (pg/mL)
IL-21 (pg/ml)
IL-22 (pg/ml)
IL-23 (pg/ml)
IL-27 (pg/ml)
IL-31 (pg/ml)
MIP3α (pg/ml)
Sup -sCD14
Sup +sCD14
B
31,9
41,8
Comp-B710-A :: CD4
Comp-U379-A :: CD8
CD4
CD8
C
TGF-β1 (pg/ml)
-sCD14
+sCD14
Unstimulated
Anti-CD3 alone
Anti-CD3 plus sCD14
Anti-CD3 plus Th17 P. Cond
7%
3%
29%
34%
25%
0.5%
9%
12.5%
2.9%
Cells (% max)
Comp-B530-A :: CFSE
CFSE
CD4+CFSElo IL-17F+
CD8+CFSElo IL-17F+

S Fig. 7

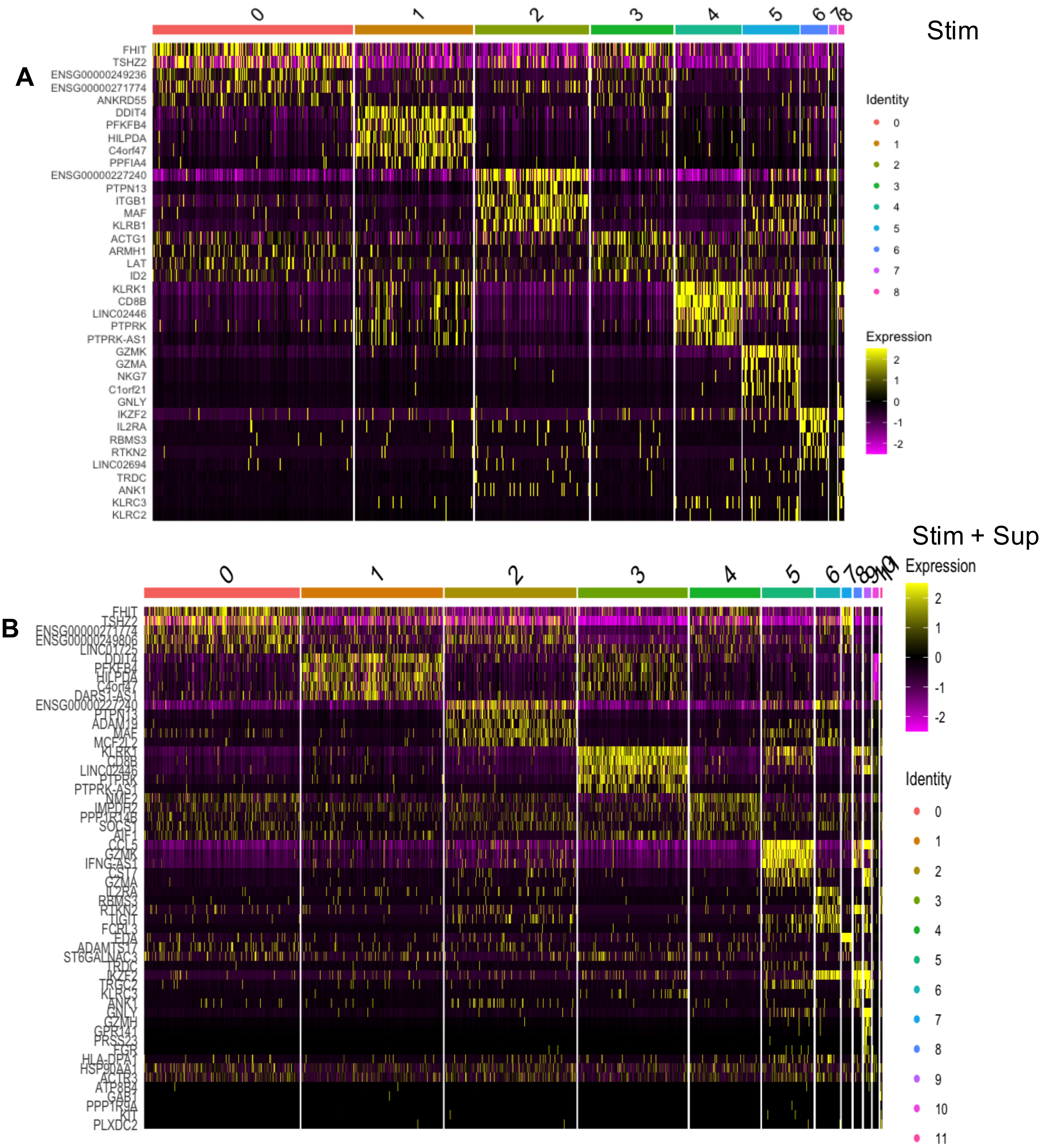

A
Stim
0
1
2
3
4
5
6
7
8
FHIT
TSHZ2
ENSG00000249236
ENSG00000271774
ANKRD55
DDIT4
PFKFB4
HILPDA
C4orf47
PPFIA4
ENSG00000227240
PTPN13
ITGB1
MAF
KLRB1
ACTG1
ARMH1
LAT
ID2
KLRK1
CD8B
LINC02446
PTPRK
PTPRK-AS1
GZMK
GZMA
NKG7
C1orf21
GNLY
IKZF2
IL2RA
RBMS3
RTKN2
LINC02694
TRDC
ANK1
KLRC3
KLRC2
Identity
0
1
2
3
4
5
6
7
8
Expression
2
1
0
-1
-2
B
Stim + Sup
0
1
2
3
4
5
6
FHIT
TSHZ2
ENSG00000271774
ENSG00000249806
LINC01725
DDIT4
PFKFB4
HILPDA
C4orf47
DARS1-AS1
ENSG00000227240
PTPN13
ADAM19
MAF
MCF2L2
KLRK1
CD8B
LINC02446
PTPRK
PTPRK-AS1
NME2
IMPDH2
PPP1R14B
SOCS1
AIF1
CCL5
GZMK
IFNG-AS1
GZMA
IL2RA
RBMS3
RTKN2
FCRL3
EDA
ADAMTS17
ST6GALNAC3
TRDC
IKZF2
TRGC2
KLRC3
ANK1
GNLY
GZMH
GPR141
PRSS23
FGR
HLA-DPA1
HSP90AA1
ACTR3
ATP8B4
GAB1
PPP1R9A
KIT
PLXDC2
Expression
2
1
0
-1
-2
Identity
0
1
2
3
4
5
6
7
8
9
10
11

S Fig. 8

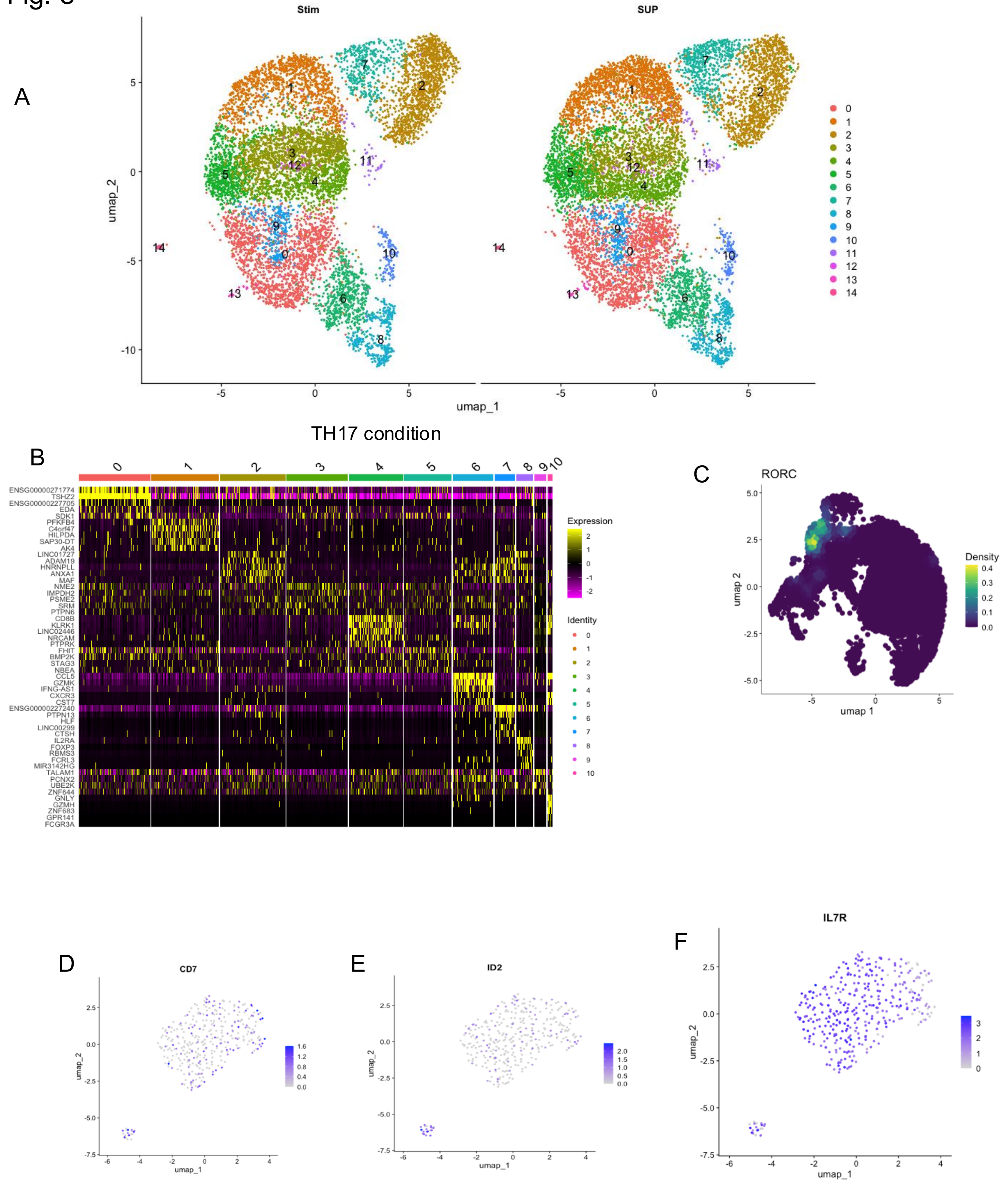
A
Stim
SUP
umap_1
umap_2
B
TH17 condition
ENSG00000271774
TSHZ2
ENSG00000227705
EDA
SDK1
PFKFB4
C4orf47
HILPDA
SAP30-DT
AK4
LINC01727
ADAM19
HNRNPLL
ANXA1
MAF
NME2
IMPDH2
PSME2
SRM
PTPN6
CD8B
KLRK1
LINC02446
NRCAM
PTPRK
FHIT
BMP2K
STAG3
NBEA
CCL5
GZMK
IFNG-AS1
CXCR3
CST7
ENSG00000227240
PTPN13
HLF
LINC00299
CTSH
IL2RA
FOXP3
RBMS3
FCRL3
MIR3142HG
TALAM1
PCNX2
UBE2K
ZNF644
GNLY
GZMH
ZNF683
GPR141
FCGR3A
Expression
Identity
C
RORC
Density
umap 1
umap 2
D
CD7
E
ID2
F
IL7R

S Fig 9

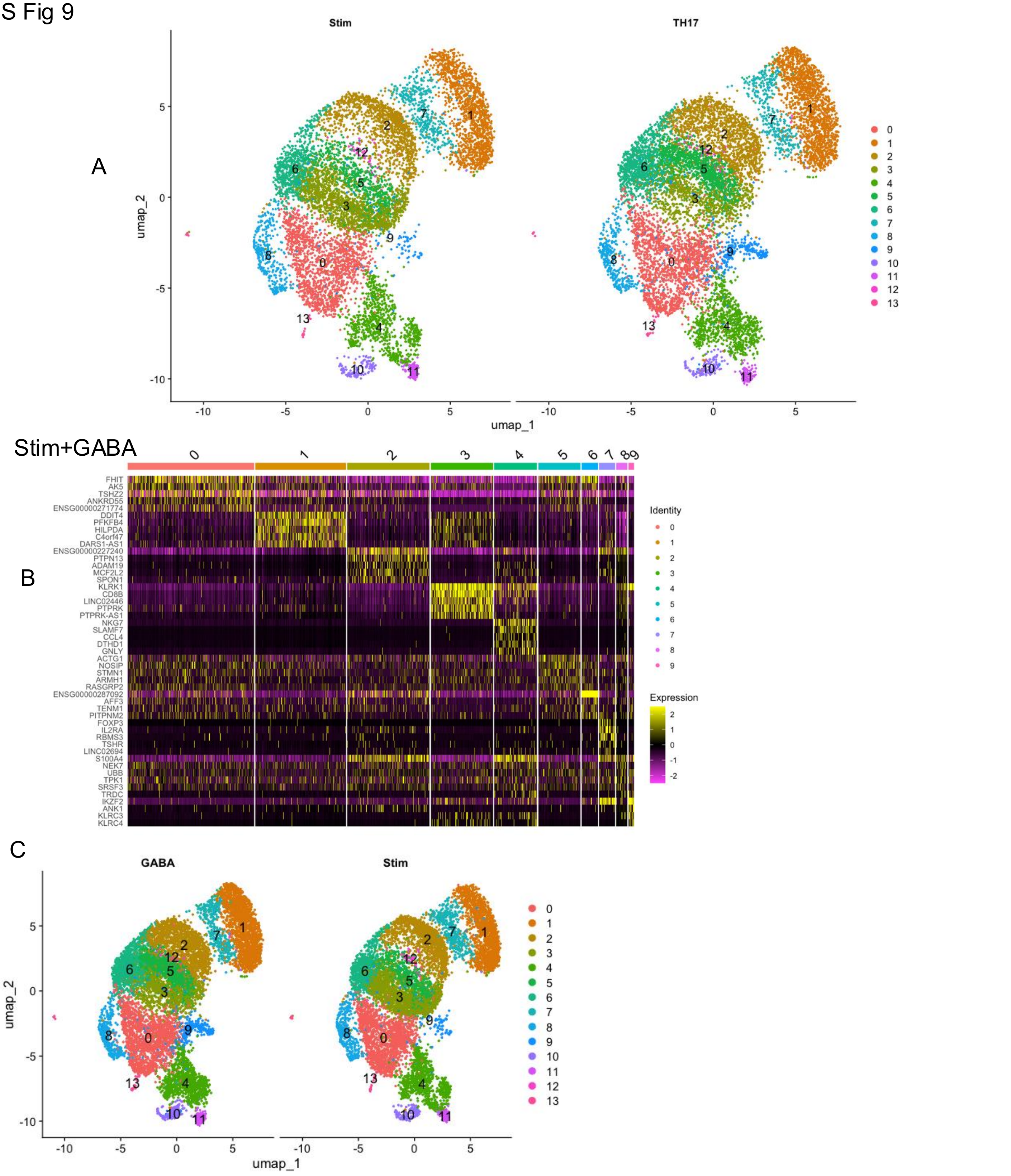
A
Stim
TH17
umap_2
umap_1
B
Stim+GABA
FHIT
AK5
TSHZ2
ANKRD55
ENSG00000271774
DDIT4
PFKFB4
HILPDA
C4orf47
DARS1-AS1
ENSG00000227240
PTPN13
ADAM19
MCF2L2
SPON1
KLRK1
CD8B
LINC02446
PTPRK
PTPRK-AS1
NKG7
SLAMF7
CCL4
DTHD1
GNLY
ACTG1
NOSIP
STMN1
ARMH1
RASGRP2
ENSG00000287092
AFF3
TENM1
PITPNM2
FOXP3
IL2RA
RBMS3
TSHR
LINC02694
S100A4
NEK7
UBB
TPK1
SRSF3
TRDC
IKZF2
ANK1
KLRC3
KLRC4
Identity
Expression
C
GABA
Stim
umap_2
umap_1
